# Changes in Body Mass Index in Children and Adolescents Living With Human Immunodeficiency Virus in Europe and Thailand Starting Dolutegravir

**DOI:** 10.1093/ofid/ofaf640

**Published:** 2025-10-11

**Authors:** The Epidemiology of Pregnancy and Paediatric Infections International Cohort Collaboration (EPPICC) Study Group, Elizabeth Chappell, Siobhan Crichton, Intira Jeannie Collins, Giorgia Dalla Valle, Charlotte Duff, Kate Edgar, Carlo Giaquinto, Charlotte Jackson, Ali Judd, Laura Mangiarini, John O’Rourke, Karen Scott, Claire Thorne, Tessa Goetghebuer, Marc Hainaut, Wivine Tremerie, Marc Delforge, Thomas Ulrik Hoffmann, Sannie Brit Nordly, Marla B Braun, Vana Spoulou, Luisa Galli, Elena Chiappini, Catiuscia Lisi, Carlotta Montagnani, Elisabetta Venturini, Magdalena Marczyńska, Jolanta Popielska, Maria Pokorska-Śpiewak, Agnieszka Ołdakowska, Konrad Zawadka, Magdalena Pluta, Małgorzata Doroba, Luminita Ene, María José Mellado, Luis Escosa, Milagros García-López Hortelano, Talía Sainz, Carlos Grasa, Paula Rodríguez, Jose Tomás Ramos, Pablo Rojo, Luis Prieto-Tato, Cristina Epalza, Alfredo Tagarro, Sara Domínguez, Álvaro Ballesteros, Marta Illán, Arantxa Berzosa, Sara Guillén, Beatriz Soto, María Luisa Navarro, Jesús Saavedra, Mar Santos, Elena Rincón, David Aguilera, Begoña Santiago, Beatriz Lázaro Martín, Andrea López Suárez, Amanda Bermejo, María Penín, Jorge Martínez, Katie Badillo, Ana Belén Jiménez, Adriana Navas, Eider Oñate, Itziar Pocheville, Elisa Garrote, Elena Colino, Olga Afonso, Jorge Gómez Sirvent, Mónica Garzón, Vicente Román, Raquel Angulo, Olaf Neth, Lola Falcón, Pedro Terol, Juan Luis Santos, Álvaro Vázquez, Begoña Carazo, Antonio Medina, Francisco Lendínez, Mercedes Ibáñez, Estrella Peromingo, María Isabel Sánchez, Beatriz Ruiz, Ana Grande, Francisco José Romero, Carlos Pérez, Alejandra Méndez, Laura Calle-Miguel, Virginia Courel del Río, Marta Pareja, Begoña Losada, Mercedes Herranz, Matilde Bustillo, Pilar Collado, José Antonio Couceiro, Leticia Vila, Consuelo Calviño, Ana Isabel Piqueras, Manuel Oltra, César Gavilán, Elena Montesinos, Marta Dapena, Beatriz Jiménez, Ana Gloria Andrés, Víctor Marugán, Carlos Ochoa, Ana Isabel Menasalvas, Eloísa Cervantes, Cristina Díez, Ignacio Bernardino, María Luisa Montes, Eulalia Valencia, Ana Delgado, Rafael Rubio, Federico Pulido, Otilia Bisbal, Alfonso Monereo Alonso, Juan Berenguer, Cristina Díez, Teresa Aldamiz, Francisco Tejerina, Juan Carlos Bernaldo de Quirós, Belén Padilla, Raquel Carrillo, Pedro Montilla, Elena Bermúdez, Maricela Valerio, Jose Sanz, Alejandra Gimeno, Miguel Cervero, Rafael Torres, Santiago Moreno, María Jesús Perez, Santos del Campo, Pablo Ryan, Jesus Troya, Jesus Sanz, Juan Losa, Rafael Gomez, Miguel Górgolas, Alberto Díaz, Sara de la Fuente, Jose Antonio Iribarren, Marıa Jose Aramburu, Lourdes Martinez, Ane Josune Goikoetxea, Sofia Ibarra, Mireia de la Peña, Víctor Asensi, Michele Hernandez, María Remedios Alemán, Ricardo Pelazas, María del Mar Alonso, Ana María López, Dácil García, Jehovana Rodriguez, Miguel Angel Cardenes, Manuel A Castaño, Francisco Orihuela, Inés Pérez, Mª Isabel Mayorga, Luis Fernando Lopez-Cortes, Cristina Roca, Silvia Llaves, Marıa Jose Rios, Jesus Rodriguez, Virginia Palomo, Juan Pasquau, Coral Garcia, Jose Hernandez, Clara Martinez, Antonio Rivero, Angela Camacho, Dolores Merino, Miguel Raffo, Laura Corpa, Elisa Martinez, Fernando Mateos, Jose Javier Blanch, Miguel Torralba, Piedad Arazo, Gloria Samperiz, Celia Miralles, Antonio Ocampo, Guille Pousada, Alvaro Mena, Marta Montero, Miguel Salavert, Maria Jose Galindo, Natalia Pretel, Joaquín Portilla, Irene Portilla, Felix Gutierrez, Mar Masia, Cati Robledano, Araceli Adsuar, Carmen Hinojosa, Begoña Monteagudo, Pablo Bachiller, Jesica Abadía, Carlos Galera, Helena Albendin, Marian Fernandez, Jose Ramon Blanco, Pere Soler-Palacín, Maria Antoinette Frick, Santiago Pérez-Hoyos, Núria López, María Méndez, Clara Carreras, Borja Guarch-Ibáñez, Teresa Vallmanya, Laura Minguell-Domingo, Olga Calavia, Lourdes García, Maite Coll, Valentí Pineda, Neus Rius, Núria Rovira, Joaquín Dueñas, Clàudia Fortuny, Anna Gamell, Antoni Noguera-Julian, Lars Navér, Nora Einarsson, Vendela Hagås, Johanna Rubin, Sandra Soeria-Atmadja, Irene Alma Abela, Karoline Aebi-Popp, Alexia Anagnostopoulos, Manuel Battegay, Marc Baumann, Enos Bernasconi, Dominique Laurent Braun, Heiner C Bucher, Alexandra Calmy, Matthias Cavassini, Angela Ciuffi, Pierre-Alex Crisinel, Katharine E A Darling, Günter Dollenmaier, Andrea Duppenthaler, Matthias Egger, Luisa Elzi, Jan Sven Fehr, Jacques Fellay, Katyuska Francini, Hansjakob Furrer, Christoph Andreas Fux, Huldrych Fritz Günthard, Anna Hachfeld, David Hans-Ulrich Haerry, Barbara Hasse, Hans Hellmuth Hirsch, Matthias Hoffmann, Irene Hösli, Michael Huber, David Jackson-Perry, Christian R Kahlert, Olivia Keiser, Thomas Klimkait, Malte Kohns, Lisa Kottanattu, Roger Dimitri Kouyos, Helen Kovari, Katharina Kusejko, Niklaus Daniel Labhardt, Karoline Leuzinger, Begoña Martinez de Tejada, Catja Marzolini, Karin Jutta Metzner, Nicolas Müller, Johannes Nemeth, Dunja Nicca, Julia Notter, Paolo Paioni, Giuseppe Pantaleo, Matthieu Perreau, Christian Polli, Elisabetta Ranieri, Andri Rauch, Luisa Paola Salazar-Vizcaya, Patrick Schmid, Olivier Segeral, Roberto F Speck, Marcel Stöckle, Philip Edward Tarr, Lecompte Marthe Than, Alexandra Trkola, Noémie Wagner, Gilles Wandeler, Maja Weisser, Sabine Yerly, Rachaneekorn Nadsasarn, Chutima Saisaengjan, Patama Deeklum, Phattharapa Khamkhen, Lucksanapon Pitikawinwong, Nattakarn Tantawarak, Pope Kosalaraksa, Chanasda Kakkaew, Tetiana Kaleeva, Yulia Baryshnikova, Iryna Raus, Olena Glutshenko, Halyna Sherstiuk, Iryna Shkurka, Natalia Delikhovska, Iryna Popova, Tetiana Golubieva, Alla Volokha, Ruslan Malyuta, Claire Thorne

**Affiliations:** London, United Kingdom

**Keywords:** antiretroviral therapy, children/adolescents, dolutegravir, HIV, weight gain

## Abstract

**Background:**

Excess weight gain has been reported in some adults on dolutegravir (DTG), but data in children/adolescents living with human immunodeficiency virus (CALHIV) are limited.

**Methods:**

CALHIV aged 2–17 years at DTG start from 15 observational cohorts across Europe and Thailand were included. Mixed models described changes in body-mass-index-for-age z-score (zBMI). We assessed (1) zBMI change 48 weeks before versus after DTG start; (2) zBMI change up to 96 weeks on DTG and associated factors; and (3) zBMI changes over 96 weeks in CALHIV aged 6–17 years at start of DTG versus protease inhibitor (PI)–based regimens using propensity score weighting.

**Results:**

Of 948 CALHIV on DTG (>99% HIV-1, <1% HIV-2), 50% were female, the median age was 13.7 (interquartile range [IQR], 11.1–15.6]) years, median zBMI was 0.31 (IQR, −0.64 to 1.19), 48% were Black, and 30% were overweight or obese at DTG start. Among 741 participants with zBMI available before and after DTG start, zBMI (95% confidence interval (CI) increased by 0.07 (.03–.11) versus 0.13 (.09–.16) (*P* = .087), in the 48 weeks before and after DTG start, respectively. Mean zBMI change by 96 weeks on DTG was 0.20 (95% CI .14–.27). In multivariable models, greatest increases in zBMI were in those aged 6–11 years at DTG start (0.34 [95% CI .23–.44]), males of “other” ethnicity (0.39 [95% CI .10–.68]), Black females (0.27 [95% CI .15–.39]), and those on tenofovir alafenamide (TAF) (0.39 [95% CI .17–.61]). There was no difference in mean zBMI change at 96 weeks among those on DTG- versus PI-based regimens (0.21 [95% CI .13–.30] vs 0.30 [95% CI .13–.48]; *P* = .354).

**Conclusions:**

CALHIV experienced zBMI increases on DTG with the largest gains in children aged 6–11 years, on TAF, with low baseline zBMI, and some variation by sex and ethnicity. However, zBMI changes over 96 weeks were comparable between those on DTG- and PI-based regimens.

## Teaser

Children and adolescents with HIV experienced increases in body-mass-index-for-age z-score (zBMI) on dolutegravir, with gains varying by age group, nucleoside reverse transcriptase inhibitor (NRTI) backbone, baseline zBMI, sex, and ethnicity. However, zBMI was also increasing before dolutegravir and was comparable to that of children/adolescents on protease inhibitor–based regimens.

Dolutegravir (DTG), a second-generation integrase strand transfer inhibitor (INSTI), is recommended as part of first-line and subsequent-line treatment for children (aged ≥4 weeks, weighing ≥3 kg), adolescents, and adults with human immunodeficiency virus (HIV) [1]. Some adult studies have reported excess weight gain and/or increased body mass index (BMI) on INSTIs as compared to other regimens [2], particularly when coadministered with tenofovir alafenamide (TAF) [3, 4]. Recent studies have highlighted the importance of accounting for the effect of prior regimens, in particular switches from tenofovir disoproxil fumarate (TDF) and efavirenz (EFV), both of which are weight-suppressing, to other antivirals that have been associated with weight gain [3, 5, 6]. There are limited data on weight gain in children and adolescents living with HIV (CALHIV) on DTG that are being rolled out globally.

The ODYSSEY trial (a randomised trial of dolutegravir [DTG]-based antiretroviral therapy versus standard of care [SOC] in children with HIV infection starting first-line or switching to second-line ART) , where CALHIV started first- or second-line antiretroviral therapy (ART), largely in sub-Saharan Africa, reported a small but statistically significantly higher weight gain (mean difference, 1 kg over 96 weeks) on DTG compared to standard of care (nonnucleoside reverse transcriptase inhibitor [NNRTI] or protease inhibitor [PI]–based regimen), which was not considered clinically significant [7]. The CHAPAS-4 trial (children with HIV in Africa – pharmacokinetics and acceptability of simple second-line antiretroviral regimens) of CALHIV starting second-line ART in sub-Saharan Africa reported increases in body-mass-index (BMI)-for-age z-score (zBMI) by 96 weeks on DTG, which was comparable to darunavir/ritonavir– or atazanavir/ritonavir–based regimens but higher than ritonavir-boosted lopinavir (LPV/r), which is thought to reflect poorer growth on LPV/r [7, 8].

Studies of growth in children on DTG from real-world settings have reported mixed results: Some reported no change in zBMI [9–11], and others reported significant increases [12–14] or increases in zBMI that were comparable to trends before DTG start [15]. Most studies have small sample sizes (n < 200), and few have explored short- and long-term growth trajectories and factors associated with zBMI increase.

The aim of this study was to explore short- and long-term zBMI changes in CALHIV on DTG in routine care compared with those on PI-based regimens.

## METHODS

Individual patient data of CALHIV who started DTG aged <18 years from 15 cohorts in 14 countries in Europe and Thailand were pooled in 2023, as part of the Epidemiology of Pregnancy and Paediatric Infections International Cohort Collaboration (EPPICC; ClinicalTrials.gov identifier NCT04677842), using a standardized data specification (hicdep.org) [16]. Data included routine demographic, clinical, and treatment-related variables throughout pediatric care and in some cohorts into adult care.

Inclusion criteria for analysis were age 2–17 years at DTG start, ≥24 weeks’ follow-up on DTG, and ≥1 zBMI measurement within 96 weeks on DTG. Children aged <2 years were excluded as BMI is less reliable in this age group [17]. CALHIV who received DTG as part of a clinical trial were excluded. Data were censored at last visit or 7 days after DTG discontinuation (defined as stopping DTG for >30 days).

zBMI is a measure of BMI relative to median BMI of children of the same age and sex, and therefore accounts for expected increases in BMI that occur as children grow. This was calculated using the British 1990 growth reference [18], where zBMI in the 2nd to <85th percentile is classified as normal weight, 85th to <95th percentile as overweight, and ≥95th percentile as obese.

### Ethics and Patient Consent Statement

This study is based on secondary analysis of routine care data of contributing cohorts. EPPICC has ethics committee approval from University College London (reference 17493/001) and all cohorts received approval or exemptions from local/national ethics committees. Some cohorts have informed consent for use of routine care data while other cohorts have a waiver of consent.

## Statistical Methods

Mixed models were used to describe zBMI changes over time (see Supplementary Table 1 for full details). Models included linear splines for time, with placement of knots (the points at which the slope of the trajectory changes) selected based on “best” model fit using Akaike information criterion. Mixed models included random intercepts for patient and slopes for time, and an exponential residual correlation structure to account for repeated measures over time.

Differences in zBMI were explored by baseline demographic and clinical characteristics at DTG start: age (2–5, 6–11, 12–17 years); sex at birth (male, female); ethnicity (Black, White, Asian, other); region of residence (United Kingdom [UK]/Ireland, Thailand, Ukraine, rest of Europe); ART/viral load (VL) status (naive, ART-experienced and suppressed [VL <200 copies/mL], ART-experienced and unsuppressed [VL 200 copies/mL], and ART-experienced with unknown VL; using nearest VL within −24/+1 week of DTG start); World Health Organization (WHO) immunosuppression for age (none/mild or advanced/severe; using nearest CD4 cell count within −24/+4 weeks) [19]; NRTI backbone (TAF, TDF, other); zBMI (nearest within −24/+4 weeks); and, among those ART-experienced, regimen immediately prior to DTG (contained any of TDF, EFV, or LPV/r vs not) and zBMI (< −1, −1 to <0, 0 to <1, ≥1).

### Change in zBMI in the 48 Weeks Before and After DTG Start

First, we compared short-term rate of zBMI change in the 48 weeks before versus after DTG start by ART/VL status in CALHIV with ≥1 zBMI measurement available in both periods. zBMI measurements from 96 weeks before to 96 weeks after DTG start were included with a linear spline with knots at −48, 0, 24, and 48 weeks used to model time. Next, we restricted the analysis to the subgroup ART-experienced/VL <200 copies/mL at DTG start, who are less likely to experience weight gain as a “return to health” effect after DTG start [20]. Univariable mixed models were used to explore whether (change in mean zBMI in first 48 weeks after DTG start) – (change in mean zBMI in 48 weeks before DTG start) differed by demographic and clinical characteristics at DTG start.

### Factors Associated With Change in zBMI Over 96 Weeks on DTG

Second, we described the long-term changes in zBMI and percentage of CALHIV moving between zBMI categories over 96 weeks on DTG. The zBMI measurements from DTG start to 96 weeks were included.

In multivariable analyses, characteristics associated with mean zBMI change, overall or with significant interactions with time on DTG (indicating rate of change in zBMI differing across groups), were identified (see Supplementary Table 1 for further details). Time since DTG start was fitted using a linear spline with knots at 0 and 24 weeks.

In the main analysis, WHO immunological stage and zBMI at DTG start were not adjusted for, due to >10% missing data. Three separate subgroup analyses were carried out using the main multivariable model: (1) adjusting for ART regimen prior to DTG among ART-experienced, (2) adjusting for WHO immunological stage among those with available data, and (3) adjusting for zBMI at DTG start among those with available data.

### Comparison of Change in zBMI Over 96 Weeks on DTG Versus PI

Third, CALHIV on DTG were compared to those on boosted PI–based regimens using data pooled in a previous EPPICC merger in 2021. Thailand was excluded from this analysis as it was not included in the 2021 merger. To maximize comparability of groups, CALHIV aged 6–17 years at start of DTG or PI combined with 2 or 3 NRTIs after 2012 were included. Propensity score weighting balanced differences in characteristics at DTG/PI start (see Supplementary Table 1 for further details). Weighted mixed models were used to compare zBMI change over 96 weeks on DTG versus PIs, overall and by ART/VL status at drug start. CALHIV on eligible DTG- and PI-based regimens were included in both groups with a patient-level random effect used to account for repeated measures.

### Sensitivity Analyses

Sensitivity analyses for the first and second analyses above explored (1) the potential impact of the coronavirus disease 2019 (COVID-19) pandemic by excluding data after January 2020 as countries such as the UK saw overall increases in overweight and obesity in children during the pandemic [21], (2) differences by NRTI backbone at DTG start excluding Ukraine and Thailand (as there was no access to TAF), and (3) zBMI trajectories using WHO growth standards/reference [17, 22]. For the second analysis, an additional sensitivity analysis was carried out restricted to CALHIV who were suppressed at DTG start.

All analyses were conducted in Stata version 18 software.

## RESULTS

### Baseline Characteristics

**Figure 1.**
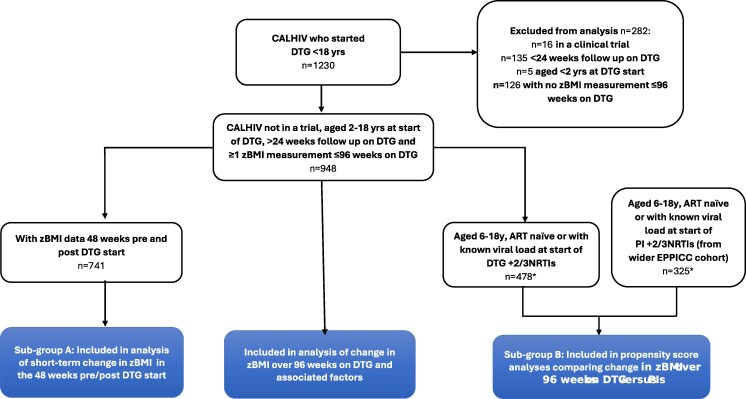
Flow diagram of children and adolescents living with human immunodeficiency virus (CALHIV) included in each analysis. *Thirty-five CALHIV contributed to both the dolutegravir and protease inhibitor groups. Abbreviations: ART, antiretroviral therapy; CALHIV, children and adolescents living with human immunodeficiency virus; DTG, dolutegravir; EPPICC, Epidemiology of Pregnancy and Paediatric Infections International Cohort Collaboration; NRTI, nucleoside reverse transcriptase inhibitor; PI, protease inhibitor; zBMI, body-mass-index-for-age z-score.

Overall, 1230 CALHIV aged <18 years started DTG, of whom 948 (77%) (>99% HIV-1, <1% HIV-2) met the inclusion criteria for analysis with ≥1 zBMI measurement in first 96 weeks on DTG (Figure 1). Half (50%) were female; 48% were Black, 32% White, 11% Asian, and 9% other ethnicity; 33% resided in the UK/Ireland, 17% Ukraine, 8% Thailand, and 41% in the rest of Europe; and 39% were born abroad (Table 1). At start of DTG, the median age was 13.7 (interquartile range [IQR], 11.1–15.6) years; 10% were treatment naive, 13% ART-experienced and unsuppressed, 51% ART-experienced and suppressed, and 25% ART-experienced with unknown viral load; and 14% had WHO advanced or severe immunosuppression for age. Seven percent started DTG on a TAF-containing regimen, 20% TDF, and 73% on other NRTIs. Among those ART-experienced at DTG start, 57% switched from LPV/r, TDF, or EFV based regimens. Characteristics by country/region are given in Supplementary Table 2.

**Table 1.** Characteristics of Children and Adolescents Living With Human Immunodeficiency Virus Included in Analyses

Characteristic	Included in Analysis of zBMI Over 96 Weeks on DTG	Included in Comparative Analysis of zBMI Change on DTG- vs PI-Based Regimens^a^	
	DTG (n = 948)	DTG (n = 467)	PI (n = 308)
Age at DTG/PI start, y	13.7 (11.1–15.6)	13.6 (11.1–15.6)	13.5 (10.9–15.3)
2–5	46 (5)	…	…
6–11	263 (28)	153 (33)	104 (34)
12–17	639 (67)	314 (67)	204 (66)
Sex			
Male	477 (50)	227 (49)	148 (48)
Female	471 (50)	240 (51)	160 (52)
Ethnicity^b^ (n = 925)			
Black	442 (48)	280 (60)	225 (73)
White	300 (32)	130 (28)	46 (15)
Asian	103 (11)	13 (3)	1 (0)
Other	80 (9)	44 (9)	36 (12)
Region			
UK/Ireland	315 (33)	57 (12)	31 (10)
Ukraine	165 (17)	223 (48)	37 (12)
Thailand	77 (8)	0 (0)	0 (0)
Rest of Europe^c^	391 (41)	187 (40)	240 (78)
ART and VL status at DTG/PI start			
Naive	99 (10)	60 (13)	97 (31)
ART-experienced and unsuppressed (VL ≥200 c/mL)	124 (13)	67 (14)	89 (29)
ART-experienced and suppressed (VL <200 c/mL)	488 (51)	340 (73)	122 (40)
ART-experienced, VL unknown	237 (25)	…	…
WHO immunological stage for age (n = 733, n = 425, n = 280)			
None/mild	629 (86)	381 (90)	201 (72)
Advanced/severe	104 (14)	44 (10)	79 (28)
Prior AIDS diagnosis (n = 938)			
AIDS-free at DTG start	744 (78)	367 (79)	250 (81)
AIDS at DTG start	194 (20)	100 (21)	58 (19)
NRTI backbone at DTG/PI start			
TAF	67 (7)	49 (10)	110 (36)
TDF	190 (20)	49 (10)	…
Other	691 (73)	369 (79)	191 (62)
Time since ART initiation, y^d^	8.9 (5.1–12.1)	8.6 (5.2–12.1)	8.3 (4.6–11.9)
ART regimen prior to DTG start^d^			
No EFV, LPV/r, or TDF	324 (43)	298 (73)	137 (65)
Contained EFV, LPV/r, or TDF	437 (57)	109 (27)	74 (35)
Previous treatment failure^d^			
No	633 (75)	318 (78)	114 (54)
Yes	211 (25)	89 (22)	97 (46)
Born abroad (n = 920)			
Yes	372 (39)	215 (46)	155 (50)
No	548 (58)	250 (54)	150 (49)
zBMI at DTG/PI start (n = 780)	0.31 (−0.64 to 1.19)	0.53 (−0.45 to 1.37)	0.39 (−0.48 to 1.16)

Data shown are No. (%) or median (interquartile range).

Abbreviations: ART, antiretroviral therapy; c/mL, copies per milliliter; DTG, dolutegravir; EFV, efavirenz; LPV/r, ritonavir-boosted lopinavir; NRTI, nucleoside reverse transcriptase inhibitor; PI, protease inhibitor; TAF, tenofovir alafenamide; TDF, tenofovir disoproxil fumarate; UK, United Kingdom; VL, viral load; WHO, World Health Organization; zBMI, body-mass-index-for-age z-score.

^a^Thirty-five children and adolescents living with human immunodeficiency virus (CALHIV) contributed to both the DTG and PI groups.

^b^Black (218 male, 224 female), White (141 male, 159 female), Asian (62 male, 41 female), other (43 male [23/43 mixed race], 37 female [28/37 mixed race]). Other ethnicity also included Hispanic, “other ethnic groups,” Maghrebian, and Roma people).

^c^Rest of Europe includes Belgium, Denmark, Germany, Greece, Italy, Poland, Romania, Spain, Sweden, and Switzerland.

^d^ART-experienced CALHIV only.

Median baseline zBMI at DTG start was 0.31 (IQR, −0.64 to 1.19), although this varied significantly across regions with markedly lower median zBMI in Ukraine and Thailand. Median duration of follow-up after DTG start was 107 (IQR, 64–173) weeks. CALHIV on DTG who were excluded from analyses due to no BMI measurement during 96 weeks on DTG (n = 126) were older and were more likely to be White, from Ukraine, have unknown viral load and on TDF at DTG start, and have shorter duration of follow-up on DTG than those included (Supplementary Table 3).

### Change in zBMI in 48 Weeks Before and After DTG Start

**Figure 2.**
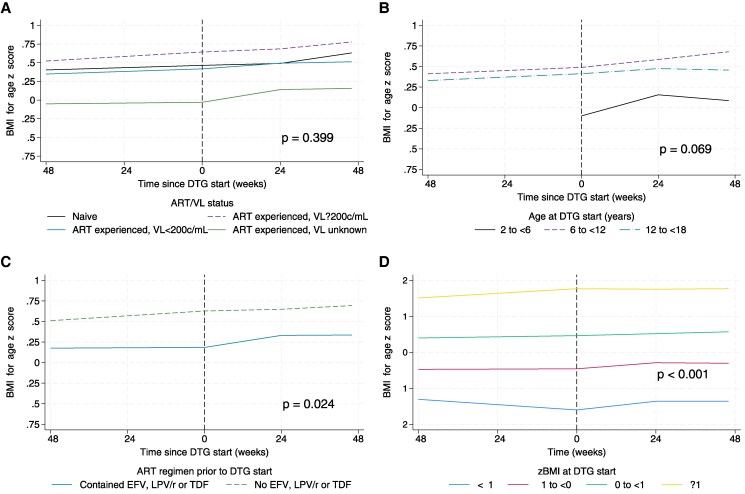
Mean body-mass-index-for-age z-score (zBMI) in the 48 weeks before and after dolutegravir (DTG) start by antiretroviral therapy (ART)/viral load (VL) status (*A*), and for children and adolescents living with human immunodeficiency virus, virally suppressed (VL <200 copies/mL) at DTG start only by age group (*B*), previous ART regimen (*C*), and zBMI at DTG start (*D*). Mean zBMI was estimated using mixed-effects models with linear splines for time since DTG start with a knot at 0 and 24 weeks, random intercept for patient, random slope for time, and correlated residuals with an exponential structure. *P* values test for differences across groups in change in zBMI over 48 weeks on DTG minus change in zBMI over 48 weeks before DTG start. zBMI was not calculated before age 2 years; therefore, the mean zBMI is not shown for the age group 2–5 years pre–DTG start in (*A*), and this category is not included in estimation of the *P* value. Abbreviations: ART, antiretroviral treatment; DTG, dolutegravir; EFV, efavirenz; LPV/r, ritonavir-boosted lopinavir; TDF, tenofovir disoproxil fumarate; VL, viral load; zBMI, body-mass-index-for-age z-score.

Of 948 CALHIV, 741 (78%) had ≥1 BMI measurement in both the 48 weeks before and after DTG start and were included in analyses of change in zBMI pre/post–DTG start (Figure 1). The zBMI increased by a mean of 0.07 (95% confidence interval [CI], .03–.11) in the 48 weeks before versus 0.13 (95% CI, .09–.16) in the 48 weeks after DTG start (*P* = .087) (Supplementary Table 5). Difference in the change in mean zBMI before versus after DTG start did not vary significantly by ART/VL status at DTG start (Figure 2*A*).

In analysis restricted to those virally suppressed at DTG start (n = 425, characteristics shown in Supplementary Table 4), the difference in the change in mean zBMI before and after DTG start varied by age (*P* = .069), previous ART regimen (*P* = .024), and baseline zBMI (*P* < .001) (Figure 2*B–D*). A significantly larger increase in zBMI in the post-DTG period (vs pre-DTG) was seen in those aged 6–11 years at DTG start (*P* = .09), among those with a prior ART regimen containing LPV/r, EFV or TDF, and those with zBMI < −1 at DTG start (*P* < .001) (Supplementary Table 5). There was no effect of sex, region, ethnicity, or NRTI backbone (Supplementary Table 5, Supplementary Figure 1).

### Factors Associated With Change in zBMI Over 96 Weeks on DTG

Among the 948 CALHIV with zBMI data in the first 96 weeks on DTG, the unadjusted change in mean zBMI by 96 weeks was 0.20 (95% CI .14–.27). Among 316 CALHIV with zBMI at both DTG start and 96 weeks, 43 (14%) were classed overweight and 53 (17%) as obese at DTG start. By 96 weeks, 15% of those classed as normal weight moved to the overweight category, and 37% classed as overweight moved to the obese category, with females more likely to develop obesity than males (Supplementary Table 6).

In multivariable analysis, there was evidence that zBMI differed across regions (*P* <.001) and by age at DTG start (*P* value for interaction [*P*_interaction_] = .002), NRTI backbone (*P*_interaction_ = .031), and ethnicity/sex (*P*_interaction_ = .09) (Supplementary Table 7). Compared to the UK/Ireland, the mean zBMI throughout follow-up was lower in other regions, by 0.64 (95% CI .31–.98) in Ukraine, 0.23 (95% CI −.33 to .80) in Thailand, and 0.21 (95% CI .02–.40) in the rest of Europe.

**Figure 3.**
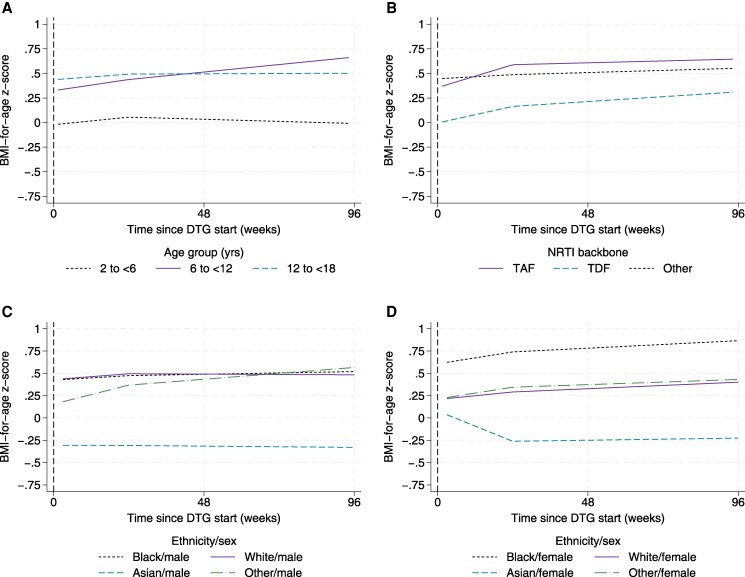
Mean body-mass-index-for-age z-score (zBMI) up to 96 weeks after dolutegravir (DTG) start by age group (*A*), nucleoside reverse transcriptase inhibitor (NRTI) backbone (*B*), ethnicity in males (*C*), and ethnicity in females (*D*). Mean zBMI (adjusted) was estimated using mixed-effects models with linear splines for time since DTG start with a knot at 24 weeks, adjusting for sex, antiretroviral therapy backbone, ethnicity, and region, with time interactions for ethnicity × sex, and NRTI backbone. Abbreviations: DTG, dolutegravir; NRTI, nucleoside reverse transcriptase inhibitor; TAF, tenofovir alafenamide; TDF, tenofovir disoproxil fumarate; zBMI, body-mass-index-for-age z-score.

The largest increases in mean zBMI were in the age group 6–11 years (Figure 3*A*) and among those starting DTG with TAF (Figure 3*B*). In terms of ethnicity and sex, the most rapid increase was among males of “other” ethnicity (Figure 3*C*, Supplementary Table 7), including those of mixed race. The next largest increase was among Black females (Figure 3*D*). In contrast, Asian females had a decrease in mean zBMI.

In subgroup analysis of ART-experienced CALHIV at DTG start (n = 744), prior ART regimen was also associated with zBMI change on DTG, independent of NRTI backbone at DTG start, with largest increases in those previously on LPV/r, TDF, or EFV-based regimens (*P*_interaction_ = .003, Supplementary Figure 2). In a second subgroup with WHO immunological stage data at DTG start (n = 621), those with advanced/severe immunosuppression had a more rapid increase in zBMI in the first 24 weeks compared to the none/mild group, but a similar rate of change after 24 weeks on DTG (*P*_interaction_ = .079) (Supplementary Figure 2).

In a third subgroup analysis of CALHIV with zBMI data at DTG start (n = 790), lower baseline zBMI was associated with larger increases in zBMI over 96 weeks (*P*_interaction_ < .001) (Supplementary Figure 3). When adjusting for zBMI at DTG start, interactions between time on DTG and age group and ethnicity/sex remained significant (*P*_interaction_ = .011 and .042, respectively) (Supplementary Figure 3). However, the interactions between time on DTG and NRTI backbone and region were no longer significant (*P*_interaction_ = .442 and .199).

### Propensity Score Analysis Comparing zBMI Change on DTG Versus PI

A subset of 467 CALHIV aged 6–11 years on 2 or 3 NRTIs + DTG regimens were compared to 308 CALHIV on 2 or 3 NRTIs + PI regimens (163 [53%] on darunavir, 98 [32%] on atazanavir, 45 [15%] on LPV, and 2 [1%] on fosamprenavir), of whom 34 contributed to both analysis groups (Figure 1, subgroup B).

**Figure 4.**
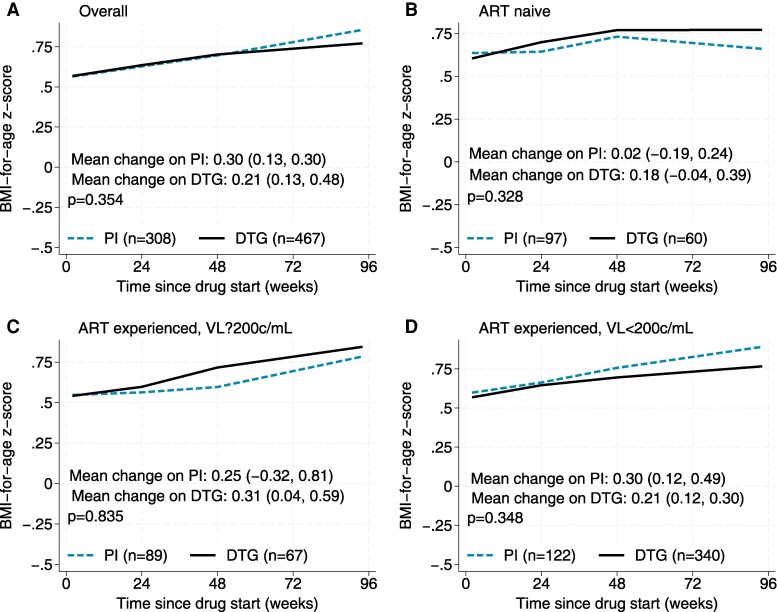
Body-mass-index-for-age z-score (zBMI) in those on dolutegravir (DTG) or protease inhibitor (PI) regimens. *A*, Overall. *B*, Antiretroviral therapy (ART) naive. *C*, ART-experienced, viral load (VL) ≥200 copies/mL. *D*, ART-experienced, VL <200 copies/mL.

Before weighting, characteristics at DTG/PI start were broadly similar, though those on DTG were more likely to start the regimen virally suppressed <200 copies/mL or to be from Ukraine (Table 1). After propensity score weighting, characteristics were well balanced, though some regional differences remained (Supplementary Table 8). Over 96 weeks, there was no difference in weighted change in mean zBMI on DTG and PI (0.21 [95% CI .13–.30] vs 0.30 [95% CI .13–.48]; *P* = .354) (Figure 4). When stratified by ART and VL status, zBMI change remained similar.

In sensitivity analyses, where we restricted calendar years of observations (to assess potential impact of the COVID-19 pandemic), excluded Ukraine and Thailand (due to no access to TAF), used WHO growth reference, and restricted to those suppressed at DTG start, findings were consistent with the main analysis (Supplementary Table 9).

## DISCUSSION

Our large study of CALHIV in routine care in Europe and Thailand included multiple approaches to assessing the short- and long-term change in zBMI on DTG. We observed weak evidence of a larger increase in mean zBMI in the 48 weeks after DTG start as compared to before DTG start (*P* = .087). There was significant evidence of larger increases in zBMI after DTG start in some subgroups such as children aged 6–11 years at DTG start. These findings persisted when restricting analyses to the subgroup virally suppressed at DTG start who are less likely to experience a “return to health” weight gain [20].

Other pediatric observational studies have reported conflicting results. A small French cohort (n = 97) reported no difference in zBMI change in 12 months pre/post–DTG start [15]. Another small study of 38 children and adolescents on INSTIs in the United States, of whom 28 were on DTG, found that zBMI increased by 0.02 (95% CI, −.09 to .13) per year prior to INSTI start, rising to 0.21 (95% CI, .08–.35) after INSTI start [13]. An Eswatini cohort (n = 460) reported significantly larger gains in zBMI after DTG start at 1.2 (95% CI 1.1–1.3) per year on DTG versus 0.3 (95% CI .2–.4) before DTG start. However the latter cohort had a low zBMI at DTG start and this may reflect a “return to health” effect, although all were virally suppressed at DTG start [12]. We observed significantly larger post-DTG increases in zBMI in those previously on LPV/r, EFV, or TDF-based regimens, and weak evidence suggesting larger increases in those starting DTG with TAF. These findings are consistent with previous studies showing the weight-suppressing effect of TDF and EFV, and poor growth among those on LPV/r, which may lead to weight gain when discontinued [5, 7 , 23 , 24]. The findings also align with previous research showing greatest weight gains associated with DTG + TAF-based regimens in adults [3, 4]. We also found significantly larger post-DTG increases in zBMI in CALHIV with low zBMI (< −1) at DTG start but not for higher zBMI groups. This aligns with evidence from DTG studies with low median zBMI that show more rapid gains [12].

Our second finding is that over 96 weeks on DTG, the largest zBMI increases were in those aged 6–11 years, males of “other” ethnicity, Black females, and those on DTG with TAF. In separate analyses of growth using EPPICC data, which compared growth by NRTI backbone in children/young people on a range of anchor drugs, we observed no differences in zBMI change in the following analyses: in the 48 weeks before versus after TAF start among those who had never been on TDF; in growth on TAF versus abacavir; and between NRTI backbones combined with DTG versus other anchor drugs [25]. However, as with this analysis, the numbers on TAF + DTG were low and may lack power to detect differences.

In our analysis, children aged 6–11 years at DTG start had the largest gains in zBMI over 96 weeks on DTG. The age effect was not observed in the ODYSSEY or CHAPAS-4 trials and has not been reported in other pediatric cohort studies, although few explored associated factors. We also observed larger zBMI gains in males of “other” ethnicity and in Black females; the latter finding is consistent with reports in adult studies [26, 27]. Among the Black female group, more than half were born abroad. There is a complex interplay between ethnicity, migrant status, socioeconomic status, and lifestyle and cultural factors, which are known to be associated with childhood obesity and are not captured here. Therefore caution is needed in interpretation of these findings and further research is warranted [28, 29].

The average zBMI increase on DTG was not large, at 0.13 over 48 weeks and 0.2 over 96 weeks. This is well below the definition of “rapid” growth, which is an increase of >0.67–1.28 over 48 weeks, which corresponds to crossing over major percentile lines traditionally shown on growth charts (ie, the 3rd, 10th, 25th, 50th, 75th, 90th, and 97th percentiles) [30–32].

Though zBMI increases were small on average, 15% of children/adolescents with normal BMI at DTG start were classified as overweight by 96 weeks. This may reflect trends in the general population [33]. In sensitivity analyses with follow-up time censored at 2020 (to take into account of potential impact of the COVID-19 pandemic) and when using WHO growth reference, similar trends in zBMI increases were observed. In these sensitivity analyses, the sex/ethnicity association was no longer significant, although the sample sizes were considerably smaller. Results also persisted when this analysis was restricted to only virally suppressed participants.

The third key finding of our study was that zBMI changes over 96 weeks on DTG were comparable to those on PI-based regimens within the same cohort, using propensity score analysis. This is consistent with findings from the CHAPAS-4 trial where DTG was not associated with excess absolute weight gain compared to darunavir- or atazanavir-based regimens, though weight gain was higher on DTG than LPV/r [7]. The ODYSSEY trial reported a small but significant change in zBMI that was 0.13 (95% CI, .01–.25) higher on DTG compared to PI/NNRTI-based regimens, but was not considered clinically significant [7].

This study has important strengths in terms of comprehensive analysis of patterns of growth and the inclusion of a large, geographically diverse sample with long duration of follow-up on DTG. However, there were important limitations. First, while we controlled for region in our analysis, there is heterogeneity between cohorts in terms of population characteristics, which are challenging to fully control for. Second, we used the British 1990 growth reference to derive z-scores. The WHO growth standards [17] and reference [22] may be more representative of the diverse population but are limited to those aged <19 years. Similar findings were observed in our sensitivity analyses using WHO references, and in previous EPPICC analyses using setting specific reference data (eg, for Thailand) [34]. Third, some CALHIV experience delayed pubertal growth spurts, which can result in a decline followed by rapid increase in zBMI [35]. For those who started ART late at older ages (eg, 6–11 years), this could result in later and more intense growth spurts [34] and potentially lead to bias. Finally, in our propensity scoring analysis of DTG- versus PI-based regimens, while weighting was used to balance clinical characteristics, we were unable to include calendar year at regimen start as there was little overlap between the calendar years in start of PIs and DTG-based regimens. Therefore, residual confounding may remain.

In conclusion, this study explored zBMI trajectories in children and adolescents on DTG using multiple methods. We observed long-term increases in zBMI over time on DTG, although this was comparable to trends observed in CALHIV on PI-based regimens. Some subgroups had greater zBMI increases on DTG, in particular those aged 6–11 years at DTG start, specific sex/ethnic groups, those who switched from TDF, EFV or LPV/r, those on TAF with DTG, and those with a low baseline zBMI < −1. Data on outcomes beyond 96 weeks are needed along with data on clinical impact of excess weight gain such as dyslipidemia, hypertension, diabetes, anxiety, and depression [36, 37]. People with HIV are at greater risk of comorbidities than the general population [38] and it is therefore important to monitor weight in these groups and screen for associated comorbidities.

## Supplementary Material

ofaf640_Supplementary_Data

## Supplementary Data

Supplementary materials are available at *Open Forum Infectious Diseases* online. Consisting of data provided by the authors to benefit the reader, the posted materials are not copyedited and are the sole responsibility of the authors, so questions or comments should be addressed to the corresponding author.

## APPENDIX

***EPPICC/Penta Coordinating Team:*** Elizabeth Chappell, Siobhan Crichton, Intira Jeannie Collins, Giorgia Dalla Valle, Charlotte Duff, Kate Edgar, Carlo Giaquinto, Charlotte Jackson, Ali Judd, Laura Mangiarini, John O’Rourke, Karen Scott, Claire Thorne.

***Collaborating cohorts:***

Belgium: Tessa Goetghebuer, Marc Hainaut, Wivine Tremerie (research nurse), Marc Delforge (data manager) (Hopital St Pierre Cohort, Brussels).

Denmark: Thomas Ulrik Hoffmann, Sannie Brit Nordly (Copenhagen).

Germany: Marla B. Braun (Frankfurt).

Greece: Vana Spoulou.

Italy: Luisa Galli, Elena Chiappini, Catiuscia Lisi (Infectious Disease Unit, Meyer Children's Hospital, IRCCS, Florence; Department of Health Sciences, University of Florence); Carlotta Montagnani, Elisabetta Venturini (Infectious Disease Unit, Meyer Children's Hospital, IRCCS, Florence).

Poland: Magdalena Marczyńska, (Head), Jolanta Popielska, Maria Pokorska-Śpiewak, Agnieszka Ołdakowska, Konrad Zawadka, Magdalena Pluta, Małgorzata Doroba (administrative assistant) (Medical University of Warsaw, Department of Children's Infectious Diseases; Hospital of Infectious Diseases, Warsaw).

Romania: Luminita Ene (“Victor Babes” Hospital Cohort, Bucharest).

Spain (Cohort of the Spanish Paediatric HIV Network - Rest of Spain [CoRISPE-S]): Pediatric Units: María José Mellado, Luis Escosa, Milagros García-López Hortelano, Talía Sainz, Carlos Grasa, Paula Rodríguez (Hospital Universitario La Paz, Madrid); Jose Tomás Ramos, Pablo Rojo, Luis Prieto-Tato, Cristina Epalza, Alfredo Tagarro, Sara Domínguez, Álvaro Ballesteros (Hospital Universitario Doce de Octubre, Madrid); Marta Illán, Arantxa Berzosa, (Hospital Clínico San Carlos, Madrid); Sara Guillén, Beatriz Soto (Hospital Universitario de Getafe, Madrid); María Luisa Navarro, Jesús Saavedra, Mar Santos, Elena Rincón, David Aguilera, Begoña Santiago, Beatriz Lázaro Martín, Andrea López Suárez (Hospital Universitario Gregorio Marañón, Madrid); Amanda Bermejo (Hospital Universitario de Móstoles, Madrid); María Penín (Hospital Universitario Príncipe de Asturias de Alcalá de Henares, Madrid); Jorge Martínez (Hospital Infantil Universitario Niño Jesús, Madrid); Katie Badillo (Hospital Universitario de Torrejón, Madrid); Ana Belén Jiménez (Hospital Fundación Jiménez Díaz, Madrid); Adriana Navas (Hospital Universitario Infanta Leonor, Madrid); Eider Oñate (Hospital Universitario Donostia, Guipúzcoa); Itziar Pocheville (Hospital Universitario Cruces, Vizcaya); Elisa Garrote (Hospital Universitario Basurto, Vizcaya); Elena Colino, Olga Afonso (Hospital Insular Materno Infantil, Gran Canaria); Jorge Gómez Sirvent (Hospital Universitario Virgen de la Candelaria, Tenerife); Mónica Garzón, Vicente Román (Hospital General, Lanzarote); Raquel Angulo (Hospital de Poniente de El Ejido, Almería); Olaf Neth, Lola Falcón (Hospital Universitario Virgen del Rocío, Sevilla); Pedro Terol (Hospital Universitario Virgen de la Macarena, Sevilla); Juan Luis Santos, Álvaro Vázquez (Hospital Universitario Virgen de las Nieves, Granada); Begoña Carazo, Antonio Medina (Hospital Regional Universitario, Málaga); Francisco Lendínez, Mercedes Ibáñez (Complejo Hospitalario Torrecárdenas, Almería); Estrella Peromingo, María Isabel Sánchez (Hospital Universitario Puerta del Mar, Cádiz); Beatriz Ruiz (Hospital Universitario Reina Sofía de Córdoba); Ana Grande (Complejo Hospitalario Universitario Infanta Cristina, Badajoz); Francisco José Romero (Complejo Hospitalario, Cáceres); Carlos Pérez, Alejandra Méndez (Hospital de Cabueñes, Asturias); Laura Calle-Miguel, Virginia Courel del Río (Hospital Universitario Central de Asturias); Marta Pareja (Complejo Hospitalario Universitario, Albacete); Begoña Losada (Hospital Virgen de la Salud, Toledo); Mercedes Herranz (Hospital Virgen del Camino, Navarra); Matilde Bustillo (Hospital Universitario Miguel Servet, Zaragoza); Pilar Collado (Hospital Clínico Universitario Lozano Blesa, Zaragoza); José Antonio Couceiro (Complejo Hospitalario Universitario, Pontevedra); Leticia Vila (Complejo Hospitalario Universitario, La Coruña); Consuelo Calviño (Hospital Universitario Lucus Augusti, Lugo); Ana Isabel Piqueras, Manuel Oltra (Hospital Universitario La Fe, Valencia); César Gavilán (Hospital Universitario de San Juan de Alicante, Alicante); Elena Montesinos (Hospital General Universitario, Valencia); Marta Dapena (Hospital General, Castellón); Beatriz Jiménez (Hospital Universitario Marqués de Valdecilla, Cantabria); Ana Gloria Andrés (Complejo Hospitalario, León); Víctor Marugán, Carlos Ochoa (Complejo Hospitalario, Zamora); Ana Isabel Menasalvas, Eloísa Cervantes (Hospital Universitario Virgen de la Arrixaca, Murcia); and Pediatric HIV-BioBank integrated in the Spanish AIDS Research Network and collaborating centers. Adult Units: Cristina Díez (Hospital Universitario Gregorio Marañón, Madrid); Ignacio Bernardino, María Luisa Montes, Eulalia Valencia, Ana Delgado (Hospital Universitario La Paz, Madrid); Rafael Rubio, Federico Pulido, Otilia Bisbal (Hospital Universitario Doce de Octubre, Madrid); Alfonso Monereo Alonso (Hospital Universitario de Getafe, Madrid); Juan Berenguer, Cristina Díez, Teresa Aldamiz, Francisco Tejerina, Juan Carlos Bernaldo de Quirós, Belén Padilla, Raquel Carrillo, Pedro Montilla, Elena Bermúdez, Maricela Valerio (Hospital Universitario Gregorio Marañón, Madrid); Jose Sanz (Hospital Universitario Príncipe de Asturias de Alcalá de Henares, Madrid); Alejandra Gimeno (Hospital Universitario de Torrejón, Madrid); Miguel Cervero, Rafael Torres (Hospital Universitario Severo Ochoa de Leganés, Madrid); Santiago Moreno, María Jesús Perez, Santos del Campo (Hospital Universitario Ramon y Cajal, Madrid); Pablo Ryan, Jesus Troya (Hospital Universitario Infanta Leonor, Madrid); Jesus Sanz (Hospital Universitario La Princesa, Madrid); Juan Losa, Rafael Gomez (Hospital Universitario Fundación Alcorcon, Madrid); Miguel Górgolas (Hospital Fundación Jiménez Díaz, Madrid); Alberto Díaz, Sara de la Fuente (Hospital Universitario Puerta de Hierro de Majadahonda, Madrid); Jose Antonio Iribarren, Marıa Jose Aramburu, Lourdes Martinez (Hospital Universitario Donostia, Guipúzcoa); Ane Josune Goikoetxea (Hospital Universitario Cruces, Vizcaya); Sofia Ibarra, Mireia de la Peña (Hospital Universitario Basurto, Vizcaya); Víctor Asensi (Hospital Universitario Central de Asturias); Michele Hernandez (Hospital Universitario Insular, Gran Canaria); María Remedios Alemán, Ricardo Pelazas, María del Mar Alonso, Ana María López, Dácil García, Jehovana Rodriguez (Hospital Universitario de Canarias, Tenerife); Miguel Angel Cardenes (Hospital Universitario Doctor Negrin, Gran Canaria); Manuel A. Castaño, Francisco Orihuela, Inés Pérez, Mª Isabel Mayorga (Hospital Regional Universitario, Málaga); Luis Fernando Lopez-Cortes, Cristina Roca, Silvia Llaves (Hospital Universitario Virgen del Rocío, Sevilla); Marıa Jose Rios, Jesus Rodriguez, Virginia Palomo (Hospital Universitario Virgen de la Macarena, Sevilla); Juan Pasquau, Coral Garcia (Hospital Universitario Virgen de las Nieves, Granada); Jose Hernandez, Clara Martinez (Hospital Universitario Clínico San Cecilio, Granada); Antonio Rivero, Angela Camacho (Hospital Universitario Reina Sofía, Córdoba); Dolores Merino, Miguel Raffo, Laura Corpa (Hospital Universitario Juan Ramon Jimenez, Huelva); Elisa Martinez, Fernando Mateos, Jose Javier Blanch (Complejo Hospitalario Universitario, Albacete); Miguel Torralba (Hospital Universitario, Guadalajara); Piedad Arazo, Gloria Samperiz (Hospital Universitario Miguel Servet, Zaragoza); Celia Miralles, Antonio Ocampo, Guille Pousada (Hospital Alvaro Cunqueiro, Pontevedra); Alvaro Mena (Complejo Hospitalario Universitario, La Coruña); Marta Montero, Miguel Salavert, (Hospital Universitario La Fe, Valencia); Maria Jose Galindo, Natalia Pretel (Hospital Clínico Universitario, Valencia); Joaquín Portilla, Irene Portilla (Hospital General Universitario, Alicante); Felix Gutierrez, Mar Masia, Cati Robledano, Araceli Adsuar (Hospital General Universitario de Elche, Alicante); Carmen Hinojosa, Begoña Monteagudo (Hospital Clínico, Valladolid); Pablo Bachiller (Hospital General, Segovia); Jesica Abadía (Hospital Universitario Rio Hortega, Valladolid); Carlos Galera, Helena Albendin, Marian Fernandez (Hospital Universitario Virgen de la Arrixaca, Murcia); Jose Ramon Blanco (Complejo Hospitalario San Millan-San Pedro, la Rioja).

Funding: T.S. received a grant from the European Society of Pediatric Infectious Diseases (ESPID Springborad Award 2023) and from the Instituto de Salud Carlos III (INT24/00011).

CoRISPE-S receives financial support from the Instituto de Salud Carlos III through the Red Temática de Investigación Cooperativa en Sida (RED RIS) and by the Centro de Investigación Biomédica en Red de Enfermedades Infecciosas-ISCIII (CIBERINFEC) [CB21/13/00025, CB21/13/00077].

Spain (Cohort of the Spanish Paediatric HIV Network - Catalonia [CoRISPE-cat]): Pere Soler-Palacín, Maria Antoinette Frick, Santiago Pérez-Hoyos (statistician) (Hospital Universitari Vall d’Hebron, Barcelona); Núria López (Hospital Universitari del Mar, Barcelona); María Méndez, Clara Carreras (Hospital Universitari Germans Trias i Pujol, Badalona); Borja Guarch-Ibáñez (Hospital Universitari JosepTrueta, Girona); Teresa Vallmanya, Laura Minguell-Domingo (Hospital Universitari Arnau de Vilanova, Lleida); Olga Calavia (Hospital Universitari Joan XXIII, Tarragona); Lourdes García (Consorci Sanitari del Maresme, Mataró); Maite Coll (Hospital General de Granollers); Valentí Pineda (Corporació Sanitària Parc Taulí, Sabadell); Neus Rius (Hospital Universitari Sant Joan, Reus); Núria Rovira (Fundació Althaia, Manresa); Joaquín Dueñas (Hospital Son Espases, Mallorca); and Clàudia Fortuny, Anna Gamell, Antoni Noguera-Julian (Hospital Sant Joan de Déu, Esplugues).

Funding: CoRISPE-cat receives financial support from the Instituto de Salud Carlos III through the Red Temática de Investigación Cooperativa en Sida (grant numbers RED RIS RD06/0006/0035 and RD06/0006/0021).

Sweden: Lars Navér, Nora Einarsson, Vendela Hagås, Johanna Rubin, Sandra Soeria-Atmadja (Karolinska University Hospital, Stockholm, Swedish InfCareHIV cohort).

Switzerland: Members of the Swiss HIV Cohort Study (SHCS) and the Swiss Mother and Child HIV Cohort (MoCHiV) Study: Irene Alma Abela, Karoline Aebi-Popp, Alexia Anagnostopoulos, Manuel Battegay, Marc Baumann, Enos Bernasconi, Dominique Laurent Braun, Heiner C. Bucher, Alexandra Calmy, Matthias Cavassini (Chairman of the Clinical and Laboratory Committee), Angela Ciuffi, Pierre-Alex Crisinel, Katharine E. A. Darling, Günter Dollenmaier, Andrea Duppenthaler, Matthias Egger, Luisa Elzi, Jan Sven Fehr, Jacques Fellay, Katyuska Francini, Hansjakob Furrer, Christoph Andreas Fux, Huldrych Fritz Günthard (President of the SHCS), Anna Hachfeld, David Hans-Ulrich Haerry (Deputy of “Positive Council”), Barbara Hasse, Hans Hellmuth Hirsch, Matthias Hoffmann, Irene Hösli, Michael Huber, David Jackson-Perry (patient representative), Christian R. Kahlert (Chairman of the Mother and Child Substudy), Olivia Keiser, Thomas Klimkait, Malte Kohns, Lisa Kottanattu, Roger Dimitri Kouyos, Helen Kovari, Katharina Kusejko (Head of Data Center), Niklaus Daniel Labhardt, Karoline Leuzinger, Begoña Martinez de Tejada, Catja Marzolini, Karin Jutta Metzner, Nicolas Müller, Johannes Nemeth, Dunja Nicca, Julia Notter, Paolo Paioni, Giuseppe Pantaleo, Matthieu Perreau, Christian Polli, Elisabetta Ranieri, Andri Rauch, Luisa Paola Salazar-Vizcaya, Patrick Schmid, Olivier Segeral, Roberto F. Speck, Marcel Stöckle, Philip Edward Tarr, Lecompte Marthe Than, Alexandra Trkola, Noémie Wagner, Gilles Wandeler (Chairman of the Scientific Board), Maja Weisser, Sabine Yerly.

Funding: This study has been financed within the framework of the SHCS, supported by the Swiss National Science Foundation (grant number 201369).

Thailand: Rachaneekorn Nadsasarn, Chutima Saisaengjan, Patama Deeklum, Phattharapa Khamkhen, Lucksanapon Pitikawinwong (Center of Excellence for Pediatric Infectious Diseases and Vaccines, Faculty of Medicine, Chulalongkorn University, Bangkok); Nattakarn Tantawarak, Pope Kosalaraksa, Chanasda Kakkaew (Infectious Disease Unit, Department of Pediatrics, Faculty of Medicine, Khon Kaen University).

Ukraine: Pediatric HIV Cohort: Tetiana Kaleeva, Yulia Baryshnikova (Odessa Regional Center for HIV/AIDS); Iryna Raus (Kiev City Center for HIV/AIDS); Olena Glutshenko (Mykolaiv Regional Center for HIV/AIDS); Halyna Sherstiuk (Dnipropetrovsk Regional Medical Center for Socially Significant Diseases); Iryna Shkurka (Center for the Prevention of HIV Infection/AIDS and Hepatitis of Chernihiv Regional Hospital); Natalia Delikhovska (Khmelnytsky Regional Center for the Prevention of HIV Infection/AIDS); Iryna Popova (Zaporizhzhia Center for the Prevention of HIV Infection/AIDS); Tetiana Golubieva (Poltava Regional Center for the Prevention of HIV Infection/AIDS); Alla Volokha (Shupyk National Healthcare University); Ruslan Malyuta (Perinatal Prevention of AIDS Initiative, Odessa).

United Kingdom: Heather . Bailey, Claire Thorne (UCL, London).

Funding: Support was provided by the Penta Foundation.
