# Supplementary material for: Changes in Body Mass Index in Children and Adolescents Living With Human Immunodeficiency Virus in Europe and Thailand Starting Dolutegravir
Source: Open Forum Infect Dis. 2025 Oct 11;12(12):ofaf640. doi: 10.1093/ofid/ofaf640 (PMC12712701; doi:10.1093/ofid/ofaf640)
Supplement: ofaf640_Supplementary_Data [file ofaf640_supplementary_data.docx]

**Supplementary material**

**Table S1: Summary of statistical methods**

| **Inclusion criteria** | **Statistical analysis** | **Subgroup Analyses** | **Sensitivity analyses** |
| --- | --- | --- | --- |
| **Change in zBMI in the 48 weeks before and after DTG start** | | | |
| - >1 zBMI 48 weeks before and >1 zBMI 48 weeks after starting DTG | - All zBMIs from 96 weeks prior to DTG start to 96 weeks after (or 7 days after DTG discontinuation if sooner) were included - Time since DTG start was fitted using a linear spline* with knots at -48, 0, 24 and 48 weeks, - Univariable mixed models** were used to explore whether (mean change in zBMI in first 48 weeks after DTG start) – (mean change in zBMI in 48 weeks before DTG start) differed across subgroups. - Characteristics considered in analyses were:   - Age at DTG start   - Ethnicity   - Region   - Sex at Birth   - ART/Viral load status at DTG start   - WHO immune status at DTG start   - ART regimen at before/after DTG start |  | Three sensitivity analyses were carried out in which   - analysis was repeated with data from start of 2020 excluded to assess the potential indirect impact of the COVID-19 pandemic - zBMI was calculated using the WHO growth standards/reference rather than the UK 1990 growth reference. CALHIV were censored at their 19^th^ birthday, the oldest age at which the WHO growth reference provides reference data. - differences by ART regimen explored in models excluding Ukraine and Thailand (where there was no TAF use reported) |
| **Change in zBMI over 96 weeks on DTG** | | | |
| - >1 zBMI in first 96 weeks after starting DTG | - All zBMIs from DTG start to 96 weeks after (or 7 days after DTG discontinuation if sooner) were included - Time since DTG start was fitted using a linear spline* with knots at 0 and 24 weeks - To explore whether the rate of change in zBMI over 96 weeks differed by demographic and clinical characteristics, interactions between each variable and time on DTG were fitted using mixed models**. - All variables with a significant association, and/or interaction (p<0.1) were considered candidates for a multivariable model, built using a backwards elimination approach, with variables with p<0.1 retained in the final model. - Characteristics considered in analyses were:   - Age at DTG start   - Ethnicity   - Region   - Sex at Birth   - ART/Viral load status at DTG start   - NRTI backbone at DTG start - As previous studies in adults have found differences in growth by sex and ethnicity ^[44]^, a three-way interaction between ethnicity, sex and time was also considered | - Among treatment-experienced CALHIV, the final model in the main analysis was additionally adjusted for ART regimen previous to DTG (included LPV/EFV/TDF vs. none of LPV/EFV/TDF) - Among CALHIV with data on WHO immune status at DTG start, the final model in the main analysis was additionally adjusted for WHO immune status - Among CALHIV with data on zBMI at DTG start, the final model in the main analysis was additionally adjusted for baseline zBMI | Four sensitivity analyses were carried out in which   - analysis was repeated with data from start of 2020 excluded to assess the potential indirect impact of the COVID-19 pandemic - zBMI was calculated using the WHO growth standards/reference rather than the UK 1990 growth reference. CALHIV were censored at their 19^th^ birthday, the oldest age at which the WHO growth reference provides reference data. - differences by ART regimen explored in models excluding Ukraine and Thailand (where there was no TAF use reported) - final model was fitted in CALHIV who were suppressed at DTG start only |
| **Change in zBMI on DTG versus PI** | | | |
| - Age 6 to <18 years at start of DTG+ 2 or 3 NRTIs or PI+2 or 3 NRTIs - PI regimen started since 2012 - >1 zBMI in first 96 weeks after starting DTG/PI - CALHIV on eligible PI and DTG regimens were included in both groups - ART-experienced CALHIV with unknown VL at regimen start were excluded due to low numbers on PI regimens | - All zBMIs from DTG/PI start to 96 weeks after (or 7 days after DTG/PI discontinuation if sooner) were included - Time since DTG/PI start was fitted using a linear spline* with knots at 0, 24 and 48 - Logistic regression models were used to estimate propensity scores (PS) defined as the probability of being on a DTG rather than a PI - A PS weight was then derived as:   *weight = 1 for those on DTG*  *PS/(1 – PS) for those on a PI*   - Weighted outcomes for those on a PI represent the treatment outcome expected if those on DTG had instead received the comparator regimen. - PSs were estimated separately by ART/Viral load status at DTG start to ensure balance within subgroups - PSs were derived based on   - age at regimen start   - sex at birth   - ethnicity,   - prior AIDS diagnosis,   - zBMI at regimen start   - TDF use at regimen start,   - time on ART (ART-experienced only)   - previous treatment failure (ART-experienced only) - Weighted mixed models*** were used to compare growth over 96 weeks on DTG and PI based regimens. - Where CALHIV were on both DTG and PI based regimen a patient level random intercept was included | - zBMI change on DTG compared to PI was assessed within subgroups defined by ART/VL status at DTG start, i.e. separately for those who were   - Naïve   - ART-experienced/ suppressed   - ART-experienced/ unsuppressed |  |
| ART = antiretroviral treatment, EFV = efavirenz, DTG = dolutegravir, LPV = ritonavir-boosted lopinavir, NRTI = nucleoside reverse transcriptase inhibitor, PI = protease inhibitor, TAF = tenofovir alafenamide fumarate, TDF = tenofovir disoproxil fumarate, UK = United Kingdom, VL = viral load, WHO = World Health Organisation, zBMI = BMI-for-age z score  *zBMI over time was modelled using linear splines, with placement of knots (points at which slope of the trajectory changes) selected based on ‘best’ model fit using Akaike's Information Criterion.  ** Mixed models included random intercepts for patient and slopes for time, as well as an exponential residual correlation structure to account for repeated measures over time.  *** Weighted mixed models included random intercepts for patient, drug (as some CALHIV contributed data on both PIs and DTG) and slopes for time. | | | |

**Table S2: Summary of CALHIV included in the study by country/region***

|  | **N** | **Age at DTG start (years)** | **Duration of follow-up (weeks)** | **zBMI (UK ref) at DTG start** | **ART backbone n (%)** | | |
| --- | --- | --- | --- | --- | --- | --- | --- |
|  |  |  |  |  | TAF | TDF | Other |
| **Belgium** | 63 | 13.6 [9.5,15.2] | 243 [115,346] | 0.77 [0.10,1.35] | 14 (22) | 6 (10) | 43 (68) |
| **Denmark** | 22 | 7.3 [6.4,11.3] | 169 [106,220] | 0.26 [-0.06,1.30] | 0 (0) | 2 (9) | 20 (91) |
| **Germany** | 4 | 13.6 [10.0,15.8] | 161 [55,270] | -1.35 [-1.38,-1.33] | 0 (0) | 0 (0) | 4 (100) |
| **Greece** | 6 | 12.7 [11.8,15.3] | 269 [146,314] | 1.89 [1.26,2.37] | 0 (0) | 0 (0) | 6 (100) |
| **Italy** | 9 | 8.3 [7.3,12.4] | 71 [37,138] | -0.04 [-0.66,0.70] | 1 (11) | 0 (0) | 8 (89) |
| **Poland** | 44 | 9.9 [7.8,13.4] | 164 [94,293] | 0.17 [-0.60,1.01] | 2 (5) | 0 (0) | 42 (95) |
| **Romania** | 3 | 14.9 [9.1,17.0] | 52 [26,65] | 0.04 [0.04,0.04] | 0 (0) | 0 (0) | 3 (100) |
| **Spain** | 149 | 13.4 [11.1,15.6] | 160 [103,229] | 0.38 [-0.66,1.33] | 2 (1) | 7 (5) | 140 (94) |
| **Sweden** | 69 | 11.7 [7.4,15.2] | 251 [196,296] | 0.07 [-0.66,1.09] | 11 (16) | 4 (6) | 54 (78) |
| **Switzerland** | 22 | 14.2 [13.1,15.9] | 102 [60,166] | 1.21 [-0.15,1.52] | 2 (9) | 9 (41) | 11 (50) |
| **Thailand** | 77 | 14.6 [8.7,16.9] | 63 [50,79] | -0.69 [-1.48,-0.03] | 0 (0) | 52 (68) | 25 (32) |
| **UK/Ireland** | 315 | 14.3 [11.7,15.9] | 96 [62,133] | 0.71 [-0.22,1.42] | 35 (11) | 26 (8) | 254 (81) |
| **Ukraine** | 165 | 13.8 [12.4,15.0] | 70 [48,106] | -0.47 [-1.09,0.34] | 0 (0) | 84 (51) | 81 (49) |
| **Total** | 948 | 13.7 [11.1,15.6] | 107 [64,173] | 0.31 [-0.64,1.19] | 67 (7) | 190 (20) | 691 (73) |

*Median [IQR] unless otherwise indicated, zBMI = BMI-for-age z score, DTG = dolutegravir, TAF = tenofovir alafenamide

**Table S3: Characteristics of patients included and excluded due to no zBMI measurements recorded in first 96 weeks on DTG**

|  | Included in analysis | Excluded from analysis | P-value |
| --- | --- | --- | --- |
|  | (n=948) | (n=126) |  |
| **Age at DTG start (years)** | 13.7 [11.1, 15.6] | 15.2 [13.1, 16.9] | <0.001 |
| 0 to <6 | 46 (5%) | 5 (4%) | <0.001 |
| 6 to <12 | 263 (28%) | 15 (12%) |  |
| 12 to <18 | 639 (67%) | 106 (84%) |  |
| **Sex (n=948, n=100)** |  |  |  |
| Male | 477 (50%) | 47 (47%) | 0.528 |
| Female | 471 (50%) | 53 (53%) |  |
| **Ethnicity (n=925)** |  |  |  |
| Black | 442 (48%) | 32 (25%) | <0.001 |
| White | 300 (32%) | 68 (54%) |  |
| Asian | 103 (11%) | 14 (11%) |  |
| Other | 80 (9%) | 12 (10%) |  |
| **Region** |  |  |  |
| UK/Ireland | 315 (33%) | 32 (25%) | <0.001 |
| Ukraine | 165 (17%) | 49 (39%) |  |
| Thailand | 77 (8%) | 11 (9%) |  |
| Rest of Europe | 391 (41%) | 34 (27%) |  |
| **ART and viral load status at start of DTG** |  |  |  |
| Naive | 99 (10%) | 5 (4%) | 0.006 |
| ART experienced, VL≥200c/mL | 124 (13%) | 20 (16%) |  |
| ART experienced, VL<200c/mL | 488 (51%) | 55 (44%) |  |
| ART experienced, VL unknown | 237 (25%) | 46 (37%) |  |
| **WHO immunological stage (n=733, n=79)** |  |  |  |
| None/mild | 629 (86%) | 72 (91%) | 0.190 |
| Advanced/severe | 104 (14%) | 7 (9%) |  |
| **Prior AIDS diagnosis** |  |  |  |
| AIDS-free at DTG start | 744 (78%) | 87 (69%) | 0.043 |
| AIDS at DTG start | 194 (20%) | 36 (29%) |  |
| **NRTI backbone** |  |  |  |
| Other | 691 (73%) | 70 (56%) | <0.001 |
| TAF | 67 (7%) | 10 (8%) |  |
| TDF | 190 (20%) | 46 (37%) |  |
| **Time since ART initiation (years)*** | 8.9 [5.1, 12.1] | 11.8 [8.3, 14.1] | <0.001 |
| **ART regimen prior to DTG start*** |  |  |  |
| No EFV, LPV or TDF | 411 (48%) | 45 (37%) | 0.021 |
| Contained EFV, LPV or TDF | 438 (52%) | 76 (63%) |  |
| **Previous treatment failure*** |  |  |  |
| No | 633 (75%) | 86 (71%) | 0.088 |
| Yes | 211 (25%) | 32 (26%) |  |
| **Born abroad (n=920)** |  |  |  |
| Yes | 372 (39%) | 29 (23%) | <0.001 |
| No | 548 (58%) | 95 (75%) |  |
| **zBMI at DTG start** | 0.31 [-0.64, 1.19] | 0.23 [-0.38, 0.95] | 0.605 |

Data shown are n (%) or median [IQR]. AIDS = Acquired Immunodeficiency Syndrome, ART = antiretroviral treatment, EFV = efavirenz, DTG = dolutegravir, LPV = ritonavir-boosted lopinavir, NRTI = nucleoside reverse transcriptase inhibitor, PI = protease inhibitor, TAF = tenofovir alafenamide fumarate, TDF = tenofovir disoproxil fumarate, UK = United Kingdom, VL = viral load, WHO = World Health Organisation, zBMI = BMI-for-age z score *ART-experienced CALHIV only.

**Table S4: Characteristics of patients included in analysis of growth pre/post DTG start (ART-experienced/virally suppressed only)**

|  | Included in analysis of growth pre/post DTG start |
| --- | --- |
|  | (n=425) |
| **Age at DTG start (years)** | 13.4 [10.3, 15.5] |
| 2 to <6 | 21 (5%) |
| 6 to <12 | 139 (33%) |
| 12 to <18 | 265 (62%) |
| **Sex** |  |
| Male | 219 (52%) |
| Female | 206 (48%) |
| **Ethnicity (n=414)** |  |
| Black | 238 (57%) |
| White | 114 (28%) |
| Asian | 25 (6%) |
| Other | 37 (9%) |
| **Region** |  |
| UK/Ireland | 174 (41%) |
| Ukraine | 43 (10%) |
| Thailand | 9 (2%) |
| Other | 199 (47%) |
| **WHO immunological stage (n=370)** |  |
| None/mild | 359 (97%) |
| Advanced/severe | 11 (3%) |
| **Prior AIDS diagnosis** |  |
| AIDS-free at DTG start | 323 (76%) |
| AIDS at DTG start | 98 (23%) |
| **NRTI backbone** |  |
| Other | 350 (82%) |
| TAF | 31 (7%) |
| TDF | 44 (10%) |
| **Time since ART initiation (years)*** | 8.9 [5.1, 12.1] |
| **ART regimen prior to DTG start*** |  |
| Contained EFV, LPV or TDF | 205 (48%) |
| No EFV, LPV or TDF | 220 (52%) |
| **Previous treatment failure* (n=424)** |  |
| No | 335 (79%) |
| Yes | 89 (21%) |
| **Born abroad (n=410)** |  |
| Yes | 187 (44%) |
| No | 223 (52%) |
| **zBMI at DTG start** | 0.39 [-0.49, 1.32] |

Data shown are n (%) or median [IQR]. AIDS = Acquired Immunodeficiency Syndrome, ART = antiretroviral treatment, EFV = efavirenz, DTG = dolutegravir, LPV = ritonavir-boosted lopinavir, NRTI = nucleoside reverse transcriptase inhibitor, PI = protease inhibitor, TAF = tenofovir alafenamide fumarate, TDF = tenofovir disoproxil fumarate, UK = United Kingdom, VL = viral load, WHO = World Health Organisation, zBMI = BMI-for-age z score. *ART-experienced CALHIV only.


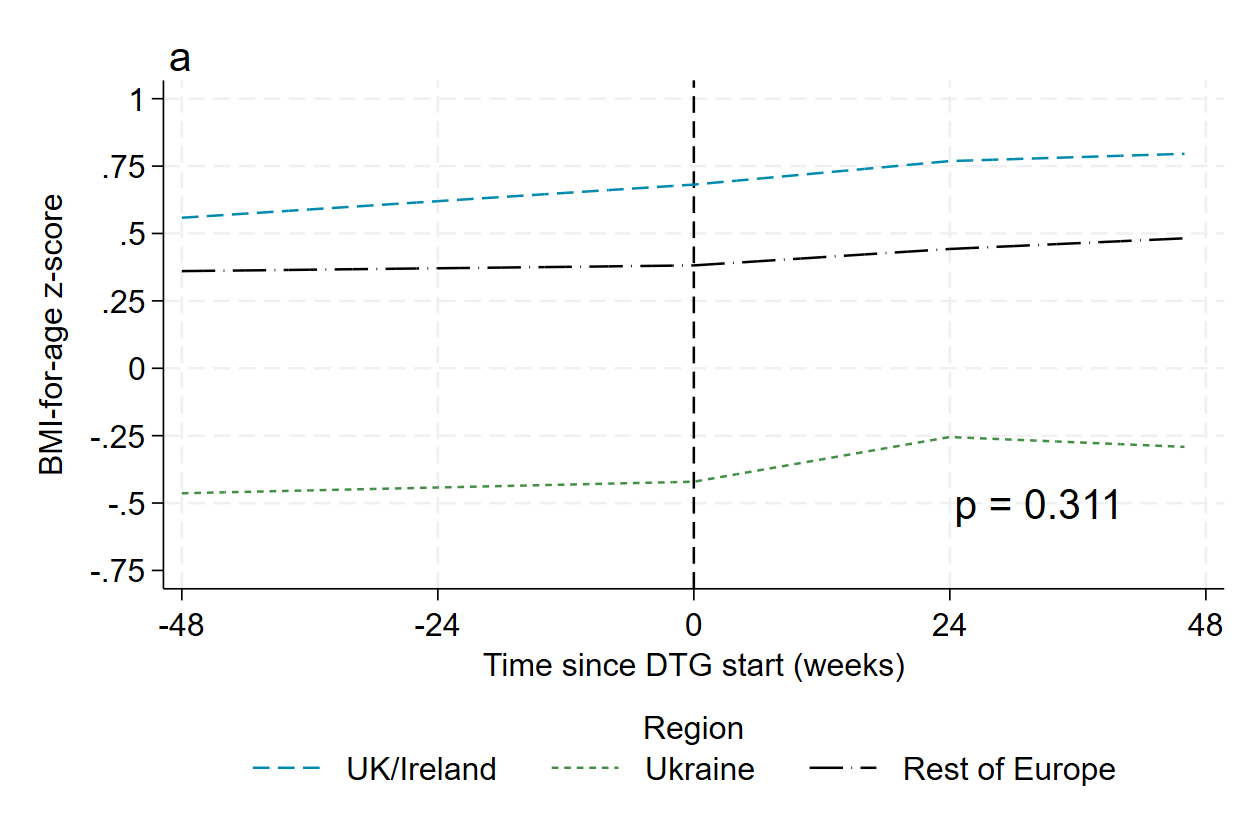

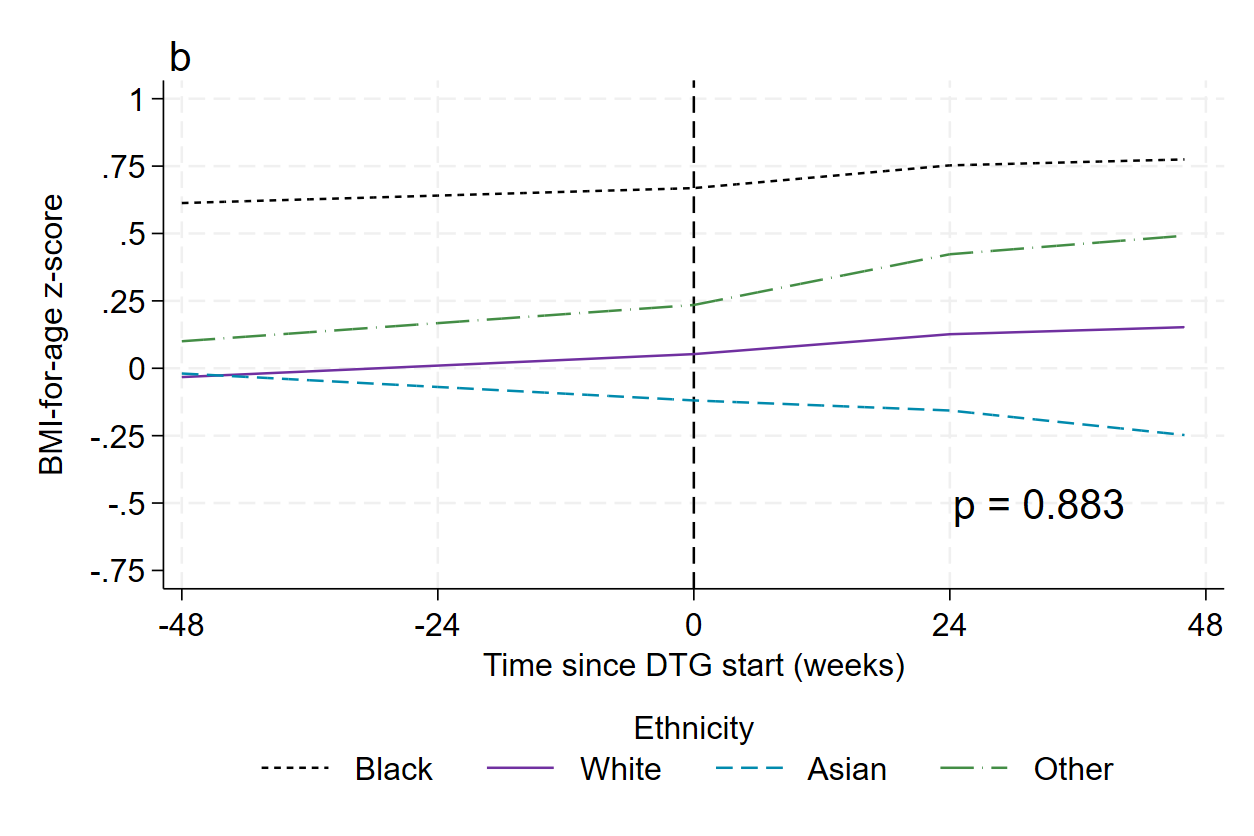


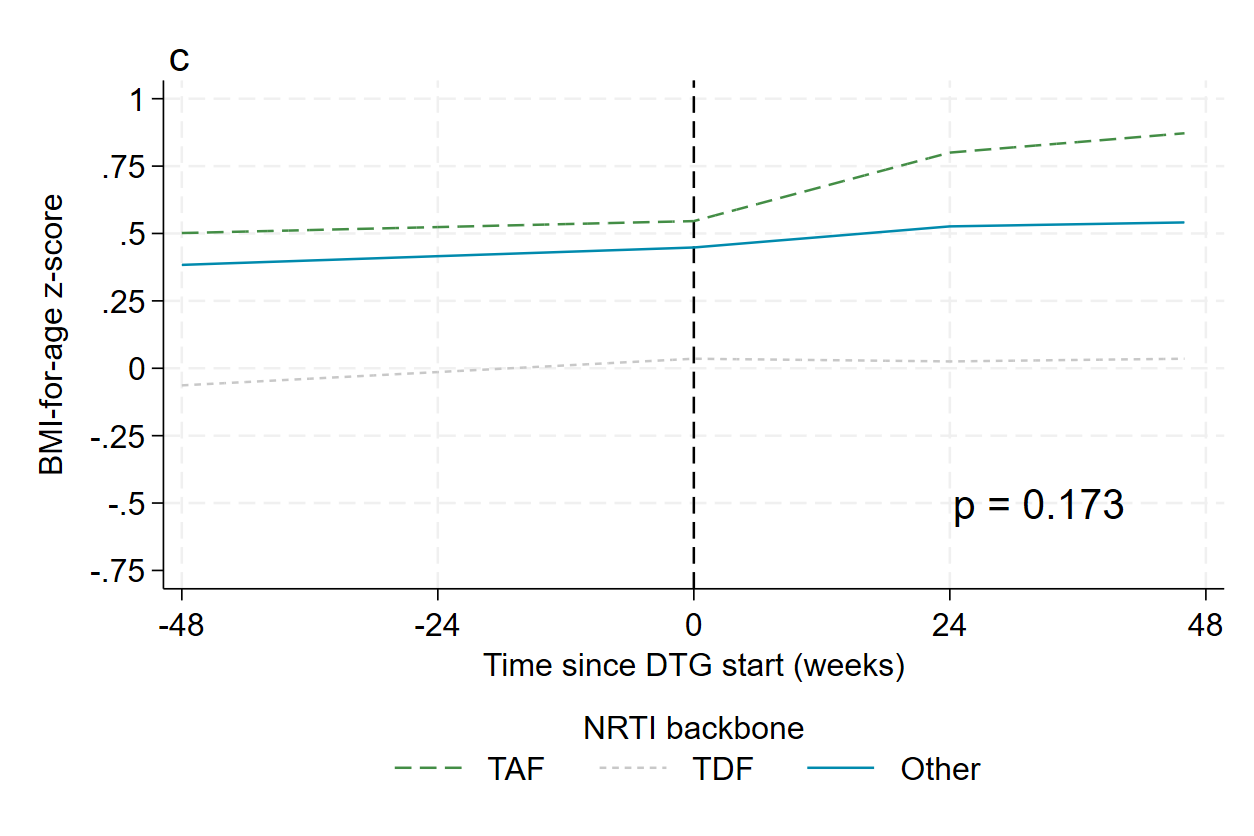

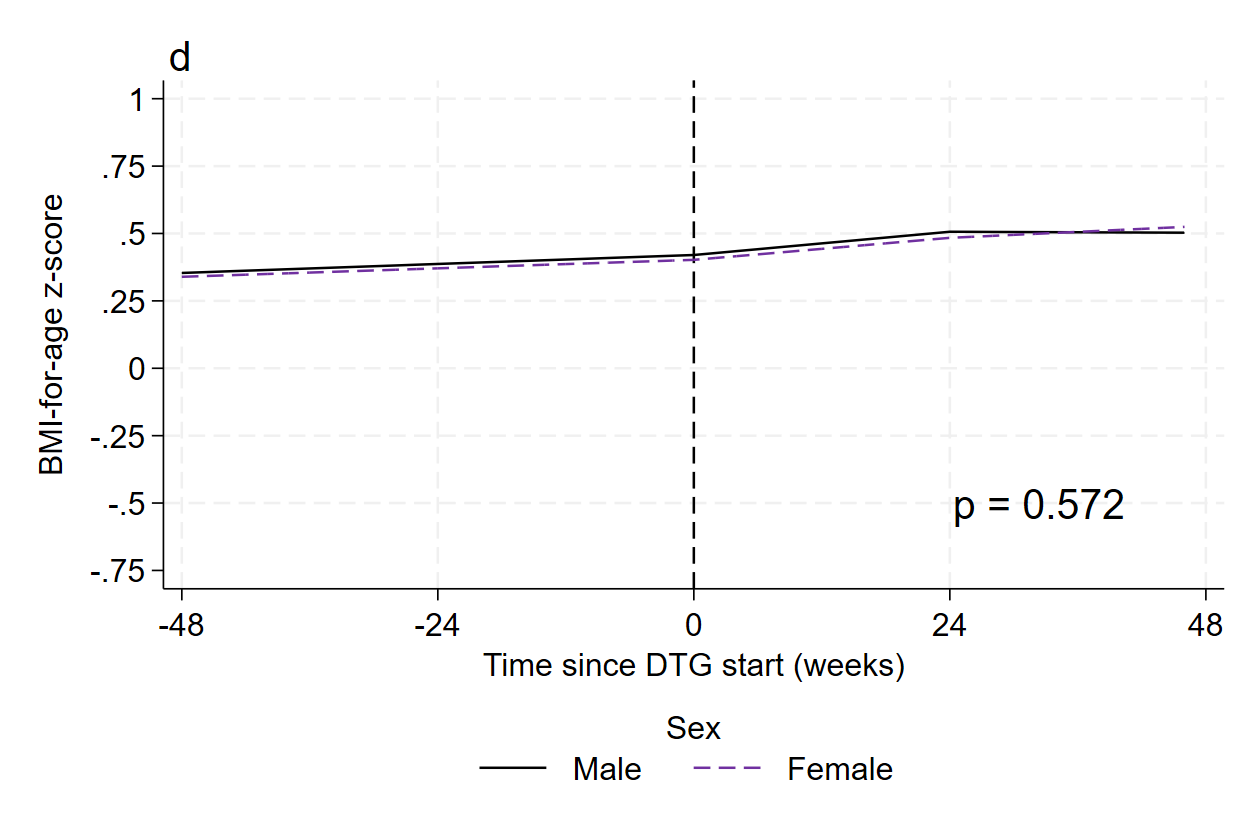


Mean BMI-for-age z-score was estimated using mixed effects models with linear splines for time since DTG start with a knot at 24 weeks. P-values test for differences across groups in change in zBMI over 48 weeks on DTG minus change in zBMI over 48 weeks before DTG start. ART = antiretroviral treatment, DTG = dolutegravir, UK = United Kingdom, VL = viral load, WHO = World Health Organisation, zBMI = BMI-for-age z score

## **Figure S1: Mean BMI-for-age z-score in the 48 weeks pre/post DTG start in CALHIV virally suppressed at DTG start only (VL<200c/mL), by a) region (Thailand excluded due to low numbers) b) ethnicity c) NRTI backbone d) sex**

## **Table S5: Comparison of unadjusted mean change in zBMI 48 weeks before and after DTG start**

|  | Total | 48 weeks pre DTG start | | 48 weeks post DTG start | | - |
| --- | --- | --- | --- | --- | --- | --- |
|  | n | Mean change | 95% CI | Mean change | 95% CI | P-value |
| **All** | 741 | 0.07 | (0.03, 0.11) | 0.13 | (0.09, 0.16) | p=0.087 |
| **ART/VL status at DTG start** |  |  |  |  |  | (p=0.399) |
| Naive | 59 | 0.06 | (-0.16, 0.28) | 0.18 | (0.04, 0.32) | p=0.408 |
| ART-experienced, VL≥200 | 96 | 0.12 | (0.01, 0.23) | 0.14 | (0.04, 0.25) | p=0.777 |
| ART-experienced, VL<200 | 425 | 0.07 | (0.02, 0.12) | 0.10 | (0.05, 0.15) | p=0.476 |
| ART-experienced, VL unknown | 161 | 0.02 | (-0.08, 0.12) | 0.19 | (0.09, 0.29) | p=0.032 |
| In ART-experienced, virally suppressed CALHIV only: | | | | | | |
| **Age at DTG start** |  |  |  |  |  | (p=0.069) |
| 6 to <12 years | 139 | 0.08 | (-0.01, 0.17) | 0.20 | (0.11, 0.28) | p=0.090 |
| 12 to <18 years | 265 | 0.08 | (0.02, 0.15) | 0.04 | (-0.03, 0.11) | p=0.440 |
| **Sex** |  |  |  |  |  | (p=0.572) |
| Male | 219 | 0.07 | (-0.01, 0.14) | 0.08 | (0.01, 0.16) | p=0.786 |
| Female | 206 | 0.06 | (-0.01, 0.14) | 0.13 | (0.05, 0.20) | p=0.296 |
| **Ethnicity** |  |  |  |  |  | (p=0.883) |
| Black | 238 | 0.06 | (-0.01, 0.12) | 0.11 | (0.04, 0.18) | p=0.346 |
| White | 114 | 0.09 | (-0.01, 0.19) | 0.10 | (0.00, 0.20) | p=0.836 |
| Asian | 25 | -0.10 | (-0.35, 0.15) | -0.14 | (-0.35, 0.07) | p=0.842 |
| Other | 37 | 0.13 | (-0.05, 0.31) | 0.26 | (0.09, 0.44) | p=0.378 |
| **Region** |  |  |  |  |  | (p=0.467) |
| UK/Ireland | 174 | 0.12 | (0.04, 0.20) | 0.12 | (0.03, 0.20) | p=0.923 |
| Ukraine | 43 | 0.04 | (-0.14, 0.22) | 0.13 | (-0.07, 0.32) | p=0.583 |
| Rest of Europe | 199 | 0.02 | (-0.05, 0.09) | 0.10 | (0.03, 0.18) | p=0.157 |
| **NRTI backbone** |  |  |  |  |  | (p=0.635) |
| TAF | 31 | 0.04 | (-0.15, 0.24) | 0.33 | (0.14, 0.53) | p=0.067 |
| TDF | 44 | 0.10 | (-0.07, 0.27) | 0.00 | (-0.17, 0.17) | p=0.482 |
| Other | 350 | 0.06 | (0.01, 0.12) | 0.09 | (0.04, 0.15) | p=0.512 |
| **ART regimen prior to DTG start** |  |  |  |  |  | (p=0.024) |
| Contained EFV, LPV or TDF | 205 | 0.01 | (-0.07, 0.08) | 0.15 | (0.08, 0.23) | p=0.019 |
| No EFV, LPV or TDF | 204 | 0.12 | (0.05, 0.19) | 0.07 | (-0.00, 0.14) | p=0.415 |
| **zBMI at DTG start** |  |  |  |  |  | (p<0.001) |
| <-1 | 54 | -0.29 | (-0.43, -0.16) | 0.24 | (0.11, 0.38) | p<0.001 |
| -1 to <0 | 100 | 0.02 | (-0.09, 0.12) | 0.15 | (0.05, 0.26) | p=0.096 |
| 0 to <1 | 111 | 0.06 | (-0.03, 0.16) | 0.11 | (0.01, 0.21) | p=0.545 |
| ≥1 | 135 | 0.26 | (0.17, 0.35) | 0.00 | (-0.09, 0.09) | p<0.001 |

Mean change is reported overall and by ART/VL status for all included CALHIV. For all other factors, mean change is reported for those ART-experienced/virally suppressed only. P-values in brackets represent global tests for differences across subgroups in the difference in the rate of change in zBMI before and after DTG start. Unbracketed p-values compare rate of change in zBMI before and after DTG start within subgroups. ART = antiretroviral treatment, EFV = efavirenz, DTG = dolutegravir, LPV = ritonavir-boosted lopinavir, NRTI = nucleoside reverse transcriptase inhibitor, PI = protease inhibitor, TAF = tenofovir alafenamide fumarate, TDF = tenofovir disoproxil fumarate, UK = United Kingdom, VL = viral load, WHO = World Health Organisation, zBMI = BMI-for-age z score

**Table S6: Change in zBMI category among those with zBMI data at 0 and 96 weeks on DTG, n (%)**

|  | **zBMI category at 96wks after DTG** | | | | |
| --- | --- | --- | --- | --- | --- |
|  | Underweight | Normal | Overweight | Obese | Total |
| **zBMI category at DTG start** |  |  |  |  |  |
| **All** |  |  |  |  |  |
| Underweight | 5 (100%) |  |  |  | 5 (100%) |
| Normal | 4 (2%) | 175 (81%) | 32 (15%) | 4 (2%) | 215 (100%) |
| Overweight |  | 14 (33%) | 13 (30%) | 16 (37%) | 43 (100%) |
| Obese |  | 2 (4%) | 6 (11%) | 45 (85%) | 53 (100%) |
| Total | 9 (3%) | 191 (60%) | 51 (16%) | 65 (21%) | 316 (100%) |
| **Male** |  |  |  |  |  |
| Underweight | 2 (100%) |  |  |  | 2 (100%) |
| Normal | 2 (2%) | 90 (83%) | 16 (15%) |  | 108 (100%) |
| Overweight |  | 7 (33%) | 8 (38%) | 6 (29%) | 21 (100%) |
| Obese |  |  | 3 (12%) | 23 (88%) | 26 (100%) |
| Total | 4 (3%) | 97 (62%) | 27 (17%) | 29 (18%) | 157 (100%) |
| **Female** |  |  |  |  |  |
| Underweight | 3 (100%) |  |  |  | 3 (100%) |
| Normal | 2 (2%) | 85 (79%) | 16 (15%) | 4 (4%) | 107 (100%) |
| Overweight |  | 7 (32%) | 5 (23%) | 10 (45%) | 22 (100%) |
| Obese |  | 2 (7%) | 3 (11%) | 22 (81%) | 27 (100%) |
| Total | 5 (3%) | 94 (59%) | 24 (15%) | 36 (23%) | 159 (100%) |

zBMI = BMI-for-age z score, DTG = dolutegravir

**Figure S2: Subgroup analyses: a) by ART regimen prior to DTG start, among those ART-experienced b) by WHO immunological stage for age, adjusted**


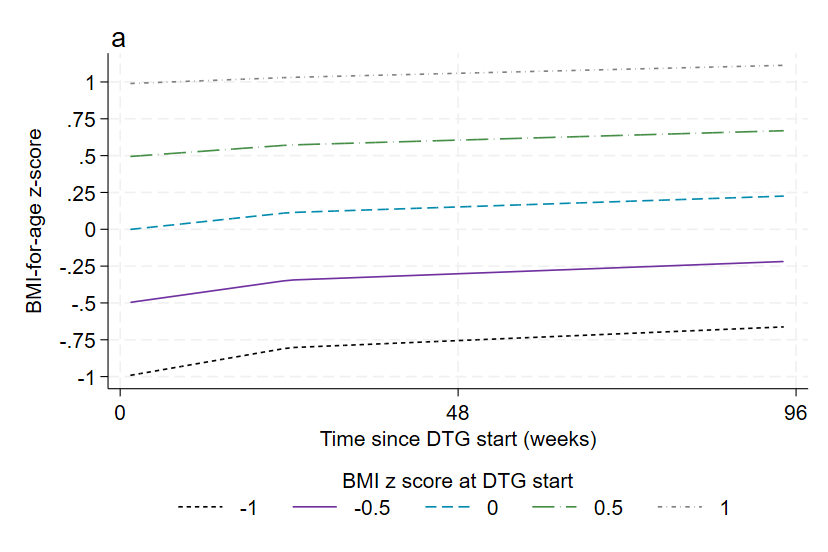

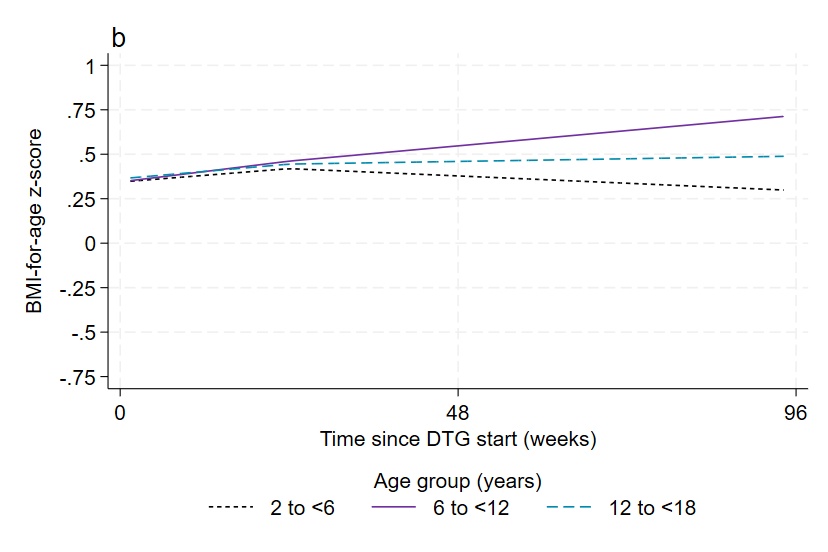

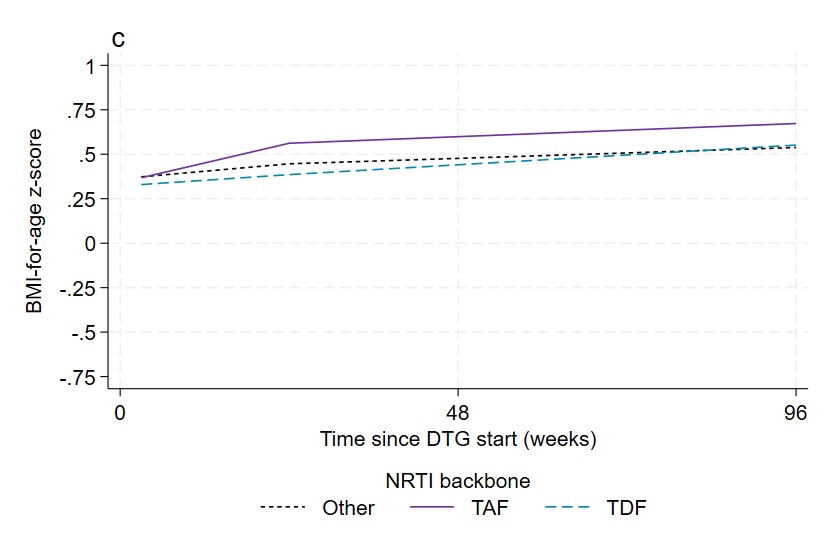


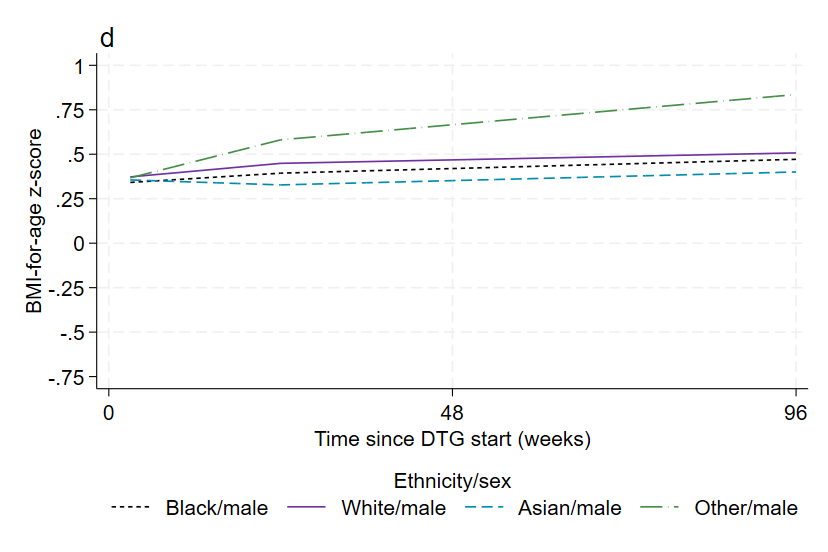

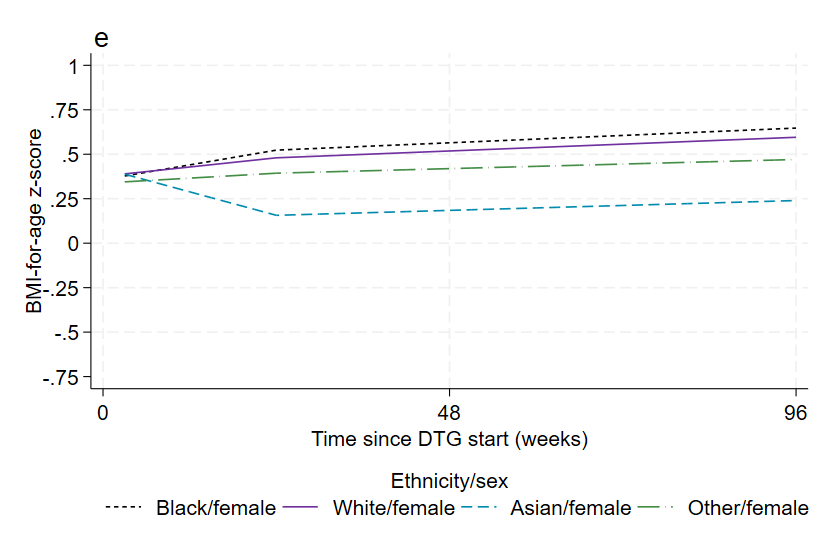


ART = antiretroviral treatment, DTG = dolutegravir, NRTI = nucleoside reverse transcriptase inhibitor, TAF = tenofovir alafenamide fumarate, TDF = tenofovir disoproxil fumarate, zBMI = BMI-for-age z score

## **Figure S3: Mean BMI-for-age z-score up to 96 weeks after DTG start by a) BMI at DTG start b) Age group c) NRTI backbone d) Ethnicity in males e) Ethnicity in females (subgroup analysis, adjusted for zBMI at DTG start).**

**Table S7: Mean change in zBMI in first 96 weeks on DTG (adjusted)**

|  | Mean change over 96 weeks | 95% CI | P-value for interaction |
| --- | --- | --- | --- |
| **Age group (years)** |  |  |  |
| 2 to <6 | -0.06 | (-0.32, 0.21) | 0.002 |
| 6 to <12 | 0.34 | (0.23, 0.44) |  |
| 12 to <18 | 0.08 | (-0.00, 0.16) |  |
| **Ethnicity*sex** |  |  |  |
| Black/male | 0.08 | (-0.04, 0.20) | 0.090 |
| Black/female | 0.27 | (0.15, 0.39) |  |
| White/male | 0.05 | (-0.12, 0.22) |  |
| White/female | 0.19 | (0.03, 0.34) |  |
| Asian/male | 0.02 | (-0.29, 0.33) |  |
| Asian/female | -0.28 | (-0.65, 0.09) |  |
| Other/male | 0.39 | (0.10, 0.68) |  |
| Other/female | 0.25 | (-0.04, 0.54) |  |
| **NRTI backbone** |  |  |  |
| TAF | 0.39 | (0.17, 0.61) | 0.031 |
| TDF | 0.22 | (0.05, 0.40) |  |
| Other | 0.11 | (0.04, 0.18) |  |

Mean BMI-for-age z-score was estimated using mixed effects models with linear splines for time since DTG start with a knot at 24 weeks, adjusting for age group, sex, NRTI backbone, ethnicity, and region, with time interactions for age group, ethnicity*sex, and NRTI backbone. DTG = dolutegravir, NRTI = nucleoside reverse transcriptase inhibitor, TAF = tenofovir alafenamide fumarate, TDF = tenofovir disoproxil fumarate, zBMI = BMI-for-age z score

**Table S8: Covariate balance before and after weighting patients on DTG and PI regimens**

|  | **DTG** | **PI** |  |  |  |
| --- | --- | --- | --- | --- | --- |
|  | **Mean ± SD or %** | **Mean ± SD or %** | **Weighted mean or %** | **Unweighted standardised difference*** | **Weighted standardised difference*** |
| **All** |  |  |  |  |  |
| **Total** | n=467 | n=308 |  |  |  |
| Male sex | 0.49±0.50 | 0.48±0.50 | 0.42 | 0.01 | 0.13 |
| Black ethnicity | 0.60±0.49 | 0.73±0.44 | 0.56 | -0.27 | 0.08 |
| Age at regimen start (years) | 13.06±3.11 | 12.99±2.99 | 13.00 | 0.02 | 0.02 |
| Prior AIDS diagnosis | 0.21±0.41 | 0.19±0.39 | 0.16 | 0.06 | 0.13 |
| Previous treatment failure | 0.22±0.41 | 0.46±0.50 | 0.21 | -0.58 | 0.03 |
| Time since ART initiation (years) | 7.49±4.97 | 5.73±5.37 | 7.29 | 0.35 | 0.04 |
| BMI-for-age z-score | 0.48±1.26 | 0.36±1.22 | 0.44 | 0.09 | 0.03 |
| ART naive | 0.13±0.33 | 0.31±0.47 | 0.12 | -0.56 | 0.02 |
| ART-experienced, VL>=200c/mL | 0.14±0.35 | 0.29±0.45 | 0.15 | -0.41 | -0.02 |
| ART-experienced, VL<200c/mL | 0.73±0.45 | 0.40±0.49 | 0.73 | 0.75 | 0.00 |
| UK & Ireland* | 0.40±0.49 | 0.78±0.42 | 0.72 | -0.77 | -0.64 |
| Ukraine* | 0.12±0.33 | 0.10±0.30 | 0.15 | 0.07 | -0.07 |
| Rest of Europe* | 0.48±0.50 | 0.12±0.33 | 0.14 | 0.71 | 0.68 |
| **Naive** |  |  |  |  |  |
| **Total** | n=60 | n=97 |  |  |  |
| Male sex | 0.45±0.50 | 0.41±0.49 | 0.44 | 0.08 | 0.02 |
| Black ethnicity | 0.73±0.45 | 0.73±0.45 | 0.75 | 0.00 | -0.04 |
| Age at regimen start (years) | 13.08±3.37 | 11.76±3.04 | 12.77 | 0.39 | 0.09 |
| Prior AIDS diagnosis | 0.08±0.28 | 0.06±0.24 | 0.08 | 0.08 | 0.02 |
| On TDF at regimen start | 0.05±0.22 | 0.22±0.41 | 0.05 | -0.76 | -0.01 |
| BMI-for-age z-score | 0.52±1.41 | 0.43±1.17 | 0.54 | 0.06 | -0.01 |
| UK & Ireland* | 0.40±0.49 | 0.70±0.46 | 0.72 | -0.61 | -0.65 |
| Ukraine* | 0.03±0.18 | 0.13±0.34 | 0.10 | -0.56 | -0.35 |
| Rest of Europe* | 0.57±0.50 | 0.16±0.37 | 0.18 | 0.80 | 0.77 |
| **ART experienced, VL≥200c/mL** |  |  |  |  |  |
| **Total** | n=67 | n=89 |  |  |  |
| Male sex | 0.33±0.47 | 0.57±0.50 | 0.29 | -0.52 | 0.09 |
| Black ethnicity | 0.49±0.50 | 0.70±0.46 | 0.43 | -0.41 | 0.13 |
| Age at regimen start (years) | 14.16±2.85 | 13.50±2.94 | 13.94 | 0.23 | 0.08 |
| Prior AIDS diagnosis | 0.24±0.43 | 0.29±0.46 | 0.19 | -0.12 | 0.11 |
| Previous treatment failure | 0.48±0.50 | 0.52±0.50 | 0.45 | -0.08 | 0.05 |
| Time since ART initiation (years) | 8.40±5.18 | 8.28±4.90 | 7.61 | 0.02 | 0.15 |
| BMI-for-age z-score | 0.47±1.22 | 0.41±1.18 | 0.33 | 0.06 | 0.12 |
| UK & Ireland* | 0.36±0.48 | 0.80±0.40 | 0.66 | -0.91 | -0.62 |
| Ukraine* | 0.24±0.43 | 0.12±0.33 | 0.22 | 0.27 | 0.05 |
| Rest of Europe* | 0.40±0.49 | 0.08±0.27 | 0.12 | 0.66 | 0.56 |
| **ART experienced, VL<200c/mL** |  |  |  |  |  |
| **Total** | n=340 | n=122 |  |  |  |
| Male sex | 0.52±0.50 | 0.47±0.50 | 0.44 | 0.11 | 0.16 |
| Black ethnicity | 0.60±0.49 | 0.75±0.43 | 0.56 | -0.32 | 0.08 |
| Age at regimen start (years) | 12.84±3.08 | 13.60±2.70 | 12.85 | -0.24 | -0.00 |
| Prior AIDS diagnosis | 0.23±0.42 | 0.21±0.41 | 0.17 | 0.05 | 0.15 |
| Previous treatment failure | 0.17±0.37 | 0.42±0.50 | 0.16 | -0.67 | 0.03 |
| Time since ART initiation (years) | 8.63±4.16 | 8.41±4.17 | 8.46 | 0.05 | 0.04 |
| BMI-for-age z-score | 0.47±1.25 | 0.27±1.27 | 0.45 | 0.16 | 0.02 |
| UK & Ireland* | 0.41±0.49 | 0.83±0.38 | 0.73 | -0.85 | -0.65 |
| Ukraine* | 0.11±0.32 | 0.06±0.23 | 0.14 | 0.18 | -0.08 |
| Rest of Europe* | 0.48±0.50 | 0.11±0.32 | 0.13 | 0.72 | 0.69 |

Logistic regression models were used to estimate propensity scores (PS) defined as the probability of being on DTG rather than a PI. A PS weight was then derived as 1 for those on DTG and PS/(1 – PS) for those on PI. Weighted outcomes for those on PIs represent the treatment outcome expected if those on DTG had instead received a PI. Standardised differences were calculated as mean on DTG minus mean on PI divided by standard deviation in the DTG group. Characteristics were considered well balanced where the standardised difference was <0.1, and adequate balance if <0.2. *All variables shown were included in the PS models apart from Region. Inclusion of region did not provide adequate balance across regions, and led to imbalance in other characteristics. AIDS = Acquired Immunodeficiency Syndrome, ART = antiretroviral treatment, DTG = dolutegravir, PI = protease inhibitor, TDF = tenofovir disoproxil fumarate, UK = United Kingdom, VL = viral load, zBMI = BMI-for-age z score

**Table S9: Summary of sensitivity analyses results**

| **Sensitivity analysis** | **Conclusions** |
| --- | --- |
| **Change in zBMI in the 48 weeks before and after DTG start** | |
| Analysis was repeated with data from start of 2020 excluded to assess the potential indirect impact of the COVID-19 pandemic | Similar results to main analysis. Mean change in zBMI among those ART-experienced and virally suppressed varied significantly by age group at DTG start and previous ART regimen. |
| zBMI was calculated using the WHO growth standards/reference rather than the UK 1990 growth reference. CALHIV were censored at their 19^th^ birthday, the oldest age at which the WHO growth reference provides reference data. | Similar results to main analysis. Mean change in zBMI among those ART-experienced and virally suppressed varied significantly by age group at DTG start and previous ART regimen. |
| Differences by ART regimen explored in models excluding Ukraine and Thailand (where there was no TAF use reported) | Similar results to main analysis. When Ukraine and Thailand were excluded, among those virally suppressed, mean difference in zBMI change before and after DTG varied significantly by previous ART regimen but not by NRTI backbone on DTG. |
| **Change in zBMI over 96 weeks on DTG** | |
| Analysis was repeated with data from start of 2020 excluded to assess the potential indirect impact of the COVID-19 pandemic | Results were similar except zBMI change over time did not differ significantly by ethnicity/sex (p=0.383). |
| zBMI was calculated using the WHO growth standards/reference rather than the UK 1990 growth reference. CALHIV were censored at their 19^th^ birthday, the oldest age at which the WHO growth reference provides reference data. | Results were similar except zBMI change over time did not differ significantly by ethnicity/sex (p=0.166). |
| Differences by ART regimen explored in models excluding Ukraine and Thailand (where there was no TAF use reported) | Greatest increase in zBMI still among those on TAF, but zBMI change over time no longer differed significantly by NRTI backbone (p=0.159). |
| Final model was fitted in CALHIV who were suppressed at DTG start only | Results were aligned with main analysis. |
